# Investigation of the hydraulic and thermal characteristics of a double concentric tubes with an inner twisted spiral tube

**DOI:** 10.1038/s41598-025-92043-3

**Published:** 2025-03-18

**Authors:** Abdalla Gomaa, Yasmin Mhrous, Mahmoud M. Abdelmagied

**Affiliations:** Department of Refrigeration and Air Conditioning Technology, Faculty of Technology and Education, Helwan University, Cairo, 11282 Egypt

**Keywords:** Enhancement of heat transfer, Heat exchanger, Twisted spiral tubes, Experimental study, Heat transfer correlation, Mechanical engineering, Energy science and technology, Engineering

## Abstract

The characteristics of heat transfer and fluid flow of twisted spiral tubes with different pitches and depths have been investigated experimentally with respect to a conventional tube (smooth tube) as a particular reference. The effects of the twisted spiral pitch ratios, *S/D*_*hy*_, depth ratios, *H/D*_*hy*_, Reynolds number and flow arrangement on the thermal performance of a twisted spiral tube heat exchanger are investigated. Three twisted spiral tubes with different pitches, *S, *of 3.9, 5.2 and 8.2 mm corresponding to twisted spiral pitch ratios, *S/D*_*hy*_, of 0.278, 0.372 and 0.586; besides three twisted spiral tubes at different depths, *H,* of 0.6, 0.95, and 1.15 mm corresponding to twisted spiral height ratio, *H/D*_*hy*_, of 0.043, 0.068 and 0.082 are experimentally examined in this study. The Reynolds number, *Re*, ranges in both inner tube side and annular side are 5000–50,000 and 1400–10,400, respectively. The results revealed that the twisted spiral pitch ratios *S/D*_*hy*_ of the 0.278achieved an enhancement of *Nu* by 38% compare to the smooth tube with a corresponding increase of 33.2% in the *f*. Also, the twisted spiral depths ratios, *H/D*_*hy*_, of 0.082 achieved higher *Nu* by 44.9%, compared to the smooth tube with a corresponding increase of 36.4% in the *f*. The thermal performance criteria reached 1.93 and 2.03 at *S/D*_*hy*_ of 0.278 and *H/D*_*hy*_ of 0.082, respectively. New correlations to expect *Nu*_*c*_ and *f*_*c*_ were predicted.

## Introduction

Double twisted spiral tube heat exchangers are used in many industries, such as industries such as oil refining automobiles, air conditioning and refrigeration, manufacturing, food production plants power plants; depended heavily on the heat exchangers. To score the optimal design of the heat exchanger, compactness, weight, cost, size and analysis of thermo-fluid characteristics (heat transfer and pressure drop) must be considered by the designers and engineers^1^. Employing heat transfer enhancement techniques in these systems allows for increased heat transfer. There are three ways to enhance the heat transfer of heat exchangers. The first method is the positive method, in which external forces are used to enhance heat transfer. The second method is the negative method, in which some modifications are made to the surfaces of the heat exchanger tubes or some additives are included in the operating fluids, such as nanofluids. The third method, which combines the negative and positive methods, is called the combined method. Many experimental and numerical studies have investigated the effects of different methods to increase heat transfer in heat exchangers. New configuration of an inner oval tube with alternating twist directions presented by Song et al.^2^. The technique is employed to improve the double-tube thermal performance. The oval tube with alternating twist directions enhances heat transfer performance compared with the conventional tubes by 173.6% for *Nu*, with an increase of 96.4% in the *f*. New correlation to predict *Nu* and *f* are provided.

The heat transfer enhancement in two-start and three start spirally corrugated tubes was numerically and experimentally studied by Kareem et al.^3,4^. The studies aimed to determine the thermal performance of two-start and three-start spirally corrugated tubes with various spiral corrugation characteristics height of 1to 3 mm and *pitch*from 9 to 25 mm. The improvement in heat transfer ranged from 21.6 to 60.5%, and the friction factor increased from 19.2 to 36.4%^3^. The spiral corrugations improved heat transfer in the range of (2.4–3.7) times that of smooth tubes. The friction factor increased approximately 1.7–2.4 times that of the smooth tube^4^. Ding et al.^5 ^investigated numerically the flow heat transfer performance of variable-direction twisted oval tubes, twisted oval tube and circular tube. The results showed that the change of the direction of tube enhance fluid mixing, inhibits the growth of the boundary layer, and enhances the heat transfer performance. The enhancement of heat transfer in six-start spirally corrugated tubes was studied by Balla^6^. The study conducted a theoretical and experimental study of smooth and corrugated tubes by studying the Nusselt number and friction coefficients. The effects of the spiral tube corrugations with various such as twisted height from 2 to 6 mm and twisted pitch from 10 to 20 mm signified on the heat transfer performance were examined. The results revealed a marked increase in heat transfer for corrugated tubes compared with smooth tubes by approximately 2.4–3.7 times, and the friction coefficient ranged from 1.7 to 2.4 times greater than that of smooth tubes. New proposal of the twisted tri-lobed tube (*TTT*) design for heat transfer enhancement is presented by Tang et al.^7^. The experimental and numerical study showed the effects of twisted tri-lobed *(TTT)* and twisted oval *(TOT) tubes* in turbulent flow on heat transfer performance. The results revealed that the heat performance improved by approximately 5.4%, whereas the friction coefficient increased by approximately 8.4%. The numerical results revealed that the friction coefficient and thermal performance were the highest when the pitch was low. The numerical study also revealed that the twisted tube with right–left hand rotation had better heat transfer performance than the tube with right hand rotation. Zhou et al.^8^ investigates the thermal hydraulic characteristics for ice slurry in a twisted tube with various twisted pitch of 125 to 300 mm. New correlations were proposed of *f* and *Nu* predict the ice slurry thermal-hydraulic performance through the twisted tube. Jin et al.^9^ studied the effects of the pitch and depth of corrugated tubes. The results showed that increasing the pitch led to a gradual decrease in heat transfer and the Nusselt number. The heat transfer increases by approximately 1.05–1.33 times compared with that of the smooth tube. A 3D numerical study on shell side heat transfer and flow characteristics of rod-baffle heat exchangers with spirally corrugated tubes was presented by Liu et al.^10^. The results revealed that the heat transfer rates for corrugated tubes (one-start, two-start, three-start, and four-start) are 104.6 −105.4%- 106.7% and 109.6%, respectively. The number of spirally corrugated tubes is 1.2 times greater than that of rod-baffle heat exchangers. A numerical study on the flow characteristics and heat transfer enhancement of oscillatory flow in a spirally corrugated tube was conducted by Xin et al.^11^. The study investigated the effect of changing the tube shape from smooth to two-start spirally corrugated. The results revealed an increase in heat transfer rates for the corrugated tube compared with the smooth tube. The thermo-fluid characteristics of double and triple tubes with various inner twisted tube design was presented by Abdelmagied^12–14^. The study investigate the impact of inner square and triangular cross-sectional profiles with various twisted pitch ratios of 6 and 9 as well as the smooth tube. The results revealed that the heat transfer coefficients of the twisted tube is greater than that of the smooth tube. New correlations to calculate *Nu* and *f* were presented.

Rozzi et al.^15^ conducted an experimental study to enhance the heat transfer and pressure loss for corrugated and smooth tubes with Newtonian and non-Newtonian fluids. The working fluids covered four fluid foods includes whole milk, cloudy orange juice, apricot and apple puree. The results showed that the heat transfer rate was greater for corrugated tubes, but the pressure decreased. Azizi et al.^16^ derive a comprehensive review of the application of twisted.

elliptical/oval tubes in heat exchangers. The study summarized the parameters that occurred to induced of the secondary flow by the fluid swirling in which disrupt the boundary layer, and intensify mixing of the fluid, in which lead to enhance the heat transfer.An experimental heat-transfer study of a heat-recovery unit made of corrugated tubes was conducted by Poredos et al.^17^. The study investigated the effects of the engineering properties of corrugated pipes with double-tube heat exchanger at various corrugation ratios of 1.4 to 3.95. The results reported that the highest heat transfer rate occurred when the corrugation ratio was less than 1.65 and that the rate of pressure drop was 3–3.5 times greater than that of the smooth tube. Xu et al.^18^ experimentally and numerically investigated the heat transfer of a heat transfer fluid in an internally four-head ribbed tube with various rib heights of 0.5 and 1 mm and pitches of 15.5–38 mm. The results revealed heat enhancement at a rate of 1.1–1.35 times from the smooth tube. The friction factor increased (by a factor of 1.3–1.5), and the pressure drop for the ribbed tubes increased more than that for the smooth tubes. Jianfeng et al.^19^ presented an experimental study on the effects of corrugated tubes and nitrate molten salt on the performance of heat transfer by using electrical energy. The study covered a corrugated groove height form 0.38 to 0.76 mm and corrugated groove pitch of 3.2 mm. The results revealed that increasing depth enhanced heat transfer as the pitch increased. New correlations were developed for both transition and turbulent flow. Asadi et al.^20^ investigated numerically the characteristics of heat transfer in a double pipe with turbulence-inducing elements. Various passive techniques were employed in which leads to better mixing of flow, develop swirl flow, and augment the rate of heat transfer. The techniques includes various geometries (smooth tube, corrugated tube, tube with spherical elements, and tube with axial fins) as well as various nano-fluids. The tube with spherical elements present the higher thermal performance among other techniques.

The heat transfer and friction characteristics of spirally corrugated tubes for outer ammonia condensation were studied experimentally by Seara and Francisco^21^. The results were compared with those of smooth tubes. The rate of heat transfer improvement factor ranged from 2.11 to 2.53, and the friction factor was 4–5 times greater than that of smooth tubes. The thermal performance of corrugated pipes is 1.27 times greater than that of smooth tubes. Turbulent heat transfer enhancement in a heat exchanger with a helically corrugated tube was studied experimentally by Pethkool et al.^22^. The study covered helical corrugated pitches ratios from 0.18 to 0.27 and corrugated heights of 0.02 to 0.06. The results revealed that the maximum heat transfer was 2.33 at *S/D*_*h*_=0.27 and *H/D*_*h*_=0.06. The Nusselt number and friction factor were 3.01 and 2.14 times greater than those of the smooth tube at low Reynolds numbers. Vicente et al.^23^ studied experimentally a comprehensive of ten corrugated tubes. The test sections were manufactured by cold rolling. The effect of rib height ratio *H/D* of 0.02–0.06 and pitch ratio *S/D* of 0.6–1.2 were the main point of the study. The results indicated that a significant impact of *S/D* and *H/D* on the thermal characteristics. New correlation to predict *Nu* and *f* were presented. Bhadouriya et al.^24^ investigate experimentally and numerically the effect of the double tube with inner twisted square duct. The effect of twist pitch ratios, annulus on *f* and heat transfer was also studied by varying the outer pipe diameter were examined. New correlation to predict *Nu* and *f* were predicted. As mention above, deforming of the twisted spiral tube of the double tube has a positive impact to enhance the characteristics of hydraulic and thermal performance of the heat exchangers as a passive method technique. A little attention has been paid (as the author knowledge) to investigate the thermal performance in the annular of twisted spiral tubes. So the current study aims to present the effect of different heat exchanger geometry parameters; such as twisted spiral pitches, depths, flow arrangements, as well as Reynolds number on the hydraulic and thermal characteristics. Three twisted spiral tubes at different *S/D*_*hy*_, of 0.278, 0.372 and 0.586 and three twisted spiral tubes at different *H/D*_*hy*_ of 0.043, 0.068 and 0.082 were designed, manufactured, and tested in both parallel and counter flow arrangements with a conventional tube heat exchanger (smooth design) as a particular references. The main concern of this study focused on enhancing the heat transfer, fluid flow characteristics, effectiveness as well as heat transfer per unit pumping power as a main point of interest. New correlations for *Nu* and *f* are derived from the results to facilitate engineering applications.

## Experimental apparatus

**Fig. 1:**
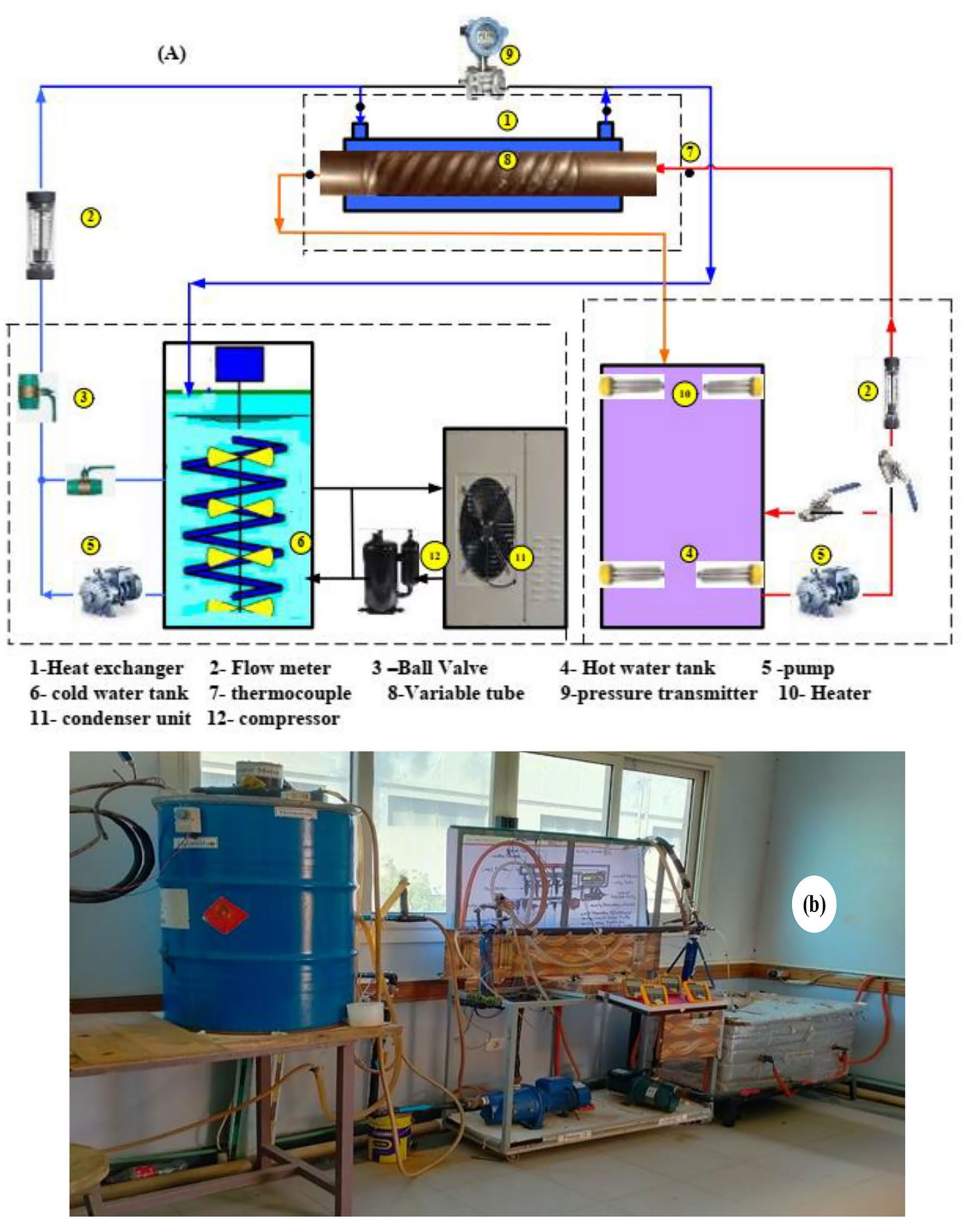
Experimental test rig, (**a**) Schematic diagram, and (**b**) photograph view.

**Fig. 2:**
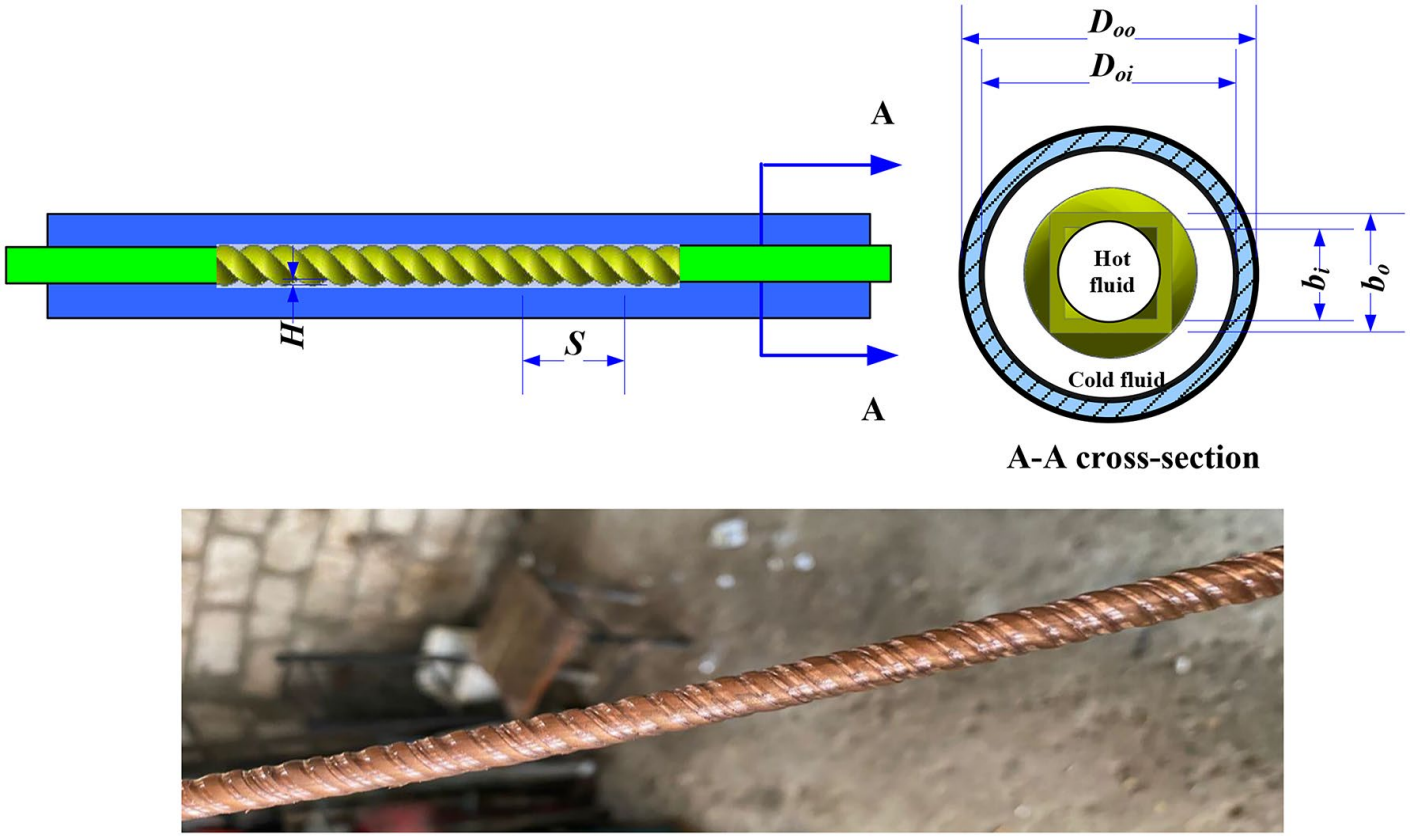
Schematic diagram of the of twisted spiral tube.

The experimental test rig consists of three main circuits: the cold water closed loop circuit, the closed loop of the hot water circuit, and the test specimen circuit, as illustrated in Fig. 1. The cold water produced from a chilled water system consists of a cooling water refrigeration circuit, an insulated tank of 0.15 m^3^ capacity, a 2HP centrifugal pump, a ball valve, and a rotameter. On the other hand, the hot water circuit consists of an insulated tank of 0.25 m^3^ capacity, four heaters of 1.5 kW each, a 1 HP centrifugal pump, and a rotameter (0–18 lit./min) to produce hot water. The temperature inside the hot water tank was maintained at 60 ± 0.5 °C. The test samples of the double concentric copper tubes with inner twisted spiral tube. The outer diameter of the external tube was 22.2 mm (7/8 inch.), and the outer diameter of the internal tube was 15.8 mm (5/8 inch.). The geometrical parameters of the twisted spiral tubes are shown in Fig. 2. The manufacturing process of the twisted spiral tube were fabricated by forming a circular straight copper tube^12^. One end of the circular tube is fixed on the lathe, and the other end of the tube is formed by cold drawing using a rotating die on the outer surface. This process produced an internal and external twisted grooves along the tube surface this leads to change the tube circular shape to square shape. Details of the dimensions of the physical geometries tubes are given in Table 1. The outer tube of the heat exchanger was thermally insulated by rubber foam insulation pipe (*k* = 0.031 W.m^−1^.K^−1^). The hot water is pumped from the hot water tank through the rotameter to the inner twisted tube of the heat exchanger, and the flow rate was adjustable by a ball valve. The cold water is also pumped from the cooling water tank through the rotameter to the annulus side of the heat exchanger, and the flow rate was adjustable by a ball valve. The temperatures of both the inner and outer fluids were measured via a K-type thermocouple with ± 0.1 °C accuracy. The pressure drop across the annulus side was measured via a pre-calibrated digital differential pressure transmitter (± 0.5 kPa accuracy).

**Table 1:** Geometric parameters of the inner twisted spiral tube (dimensions in mm).

Specimen No.	*S*	*S/D*	*H*	*H* */D*	*D*_*i, o*_	*D*_*o, o*_	*L*
1	3.9	0.278	0.95	0.068	15.8	22.2	1000
2	5.2	0.372	0.95	0.068	15.8	22.2	1000
*3*	*8.2*	*0.586*	*0.95*	*0.068*	*15.8*	*22.2*	*1000*
*4*	*5.2*	*0.372*	*0.6*	*0.043*	*15.8*	*22.2*	*1000*
*5*	*5.2*	*0.372*	*1.15*	*0.082*	*15.8*	*22.2*	*1000*
*6 (smooth tube)*	*-––*	*–––*	*–––*	*–––*	*15.8*	*22.2*	*1000*

## Data reduction

The data reduction of the measured results is summarized in the following procedures.

The heat transferred to the cold water in the test section *(Q*_*c*_*)* can be calculated as:

1 $${Q_c}={\mathop m\limits^{.} _c}C{p_c}({T_{c,o}} - {T_{c,i}})$$

The heat transferred from the hot water *(Q*_*h*_*)* can be calculated as follows:

2 $${Q_h}={\mathop m\limits^{.} _h}C{p_h}({T_{h,o}} - {T_{h,i}})$$

At zero heat loss, the amount of heat transfer on the cold side is equal to the amount of heat transfer on the hot side. To minimize the error in the calculation of the average heat transfer rate, *Q*_*ave*_ is used in the calculation of the overall heat transfer coefficient, in which the average heat transfer *Q*_*avg*_ is as follows:

3 $${\mathop Q\limits^{.} _{avg}}=0.5({\mathop Q\limits^{.} _{_{{_{c}}}}}+{\mathop Q\limits^{.} _{_{h}}})$$

The overall heat transfer coefficient, *U*_*o*_, was obtained from:

4 $${U_o}=\frac{{{{\mathop Q\limits^{.} }_{avg}}}}{{A\Delta {T_{LMTD}}}}$$

where *ΔT*_*LMTD*_ is the logarithmic mean temperature difference.

5 $$\Delta {T_{LMTD}}=\frac{{({T_{h,o}} - {T_{c,o}}) - ({T_{h,i}} - {T_{c,i}})}}{{Ln[({T_{h,o}} - {T_{c,o}})/({T_{h,i}} - {T_{c,i}})]}}$$

The Nusselt number of the inner tube was calculated from *Naphon* et al.^25^. The correlation between the Nusselt number and the Reynolds number, Prandtl number, pitch and depth of the twisted spiral is expressed as:

6 $$N{u_h}=44.26{(H/D)^{0.89}}{(S/D)^{ - 0.96}}{(R{e_h} - 1500)^{0.27}}Pr_{h}^{{ - 0.26}}$$

The correlation for the Nusselt number for smooth tubes was calculated from Naphon et al.^25^:

7 $$N{u_h}=1.84{(R{e_h} - 1500)^{0.32}}Pr_{h}^{{0.07}}$$

The Reynolds number of the hot water flow inside the tube is given by

8 $$Re=\frac{{\rho V{D_{hy,o}}}}{\mu }$$

The annulus heat transfer coefficient, *h*_*o*_, of the double twisted spiral tube heat exchanger was determined from the overall heat transfer coefficient relationship:

9 $$\frac{1}{{{U_o}}}=\frac{{{A_o}}}{{{A_i}{h_i}}}+\frac{{{A_o}\ln ({D_o}/{D_i})}}{{2\pi kL}}+\frac{1}{{{h_o}}}$$

The Nusselt number can be calculated as:

10 $$N{u_o}=\frac{{{h_o}{D_{hy,o}}}}{{{k_o}}}$$

The friction factor, *f*, can be calculated as follows:

11 $${f_o}=\frac{{2\Delta {P_o}{D_{hy,o}}}}{{L\rho {V_o}^{2}}}$$

To calculate the effectiveness of the heat exchanger, the following equation was used.

12 $$\varepsilon =\frac{{{{\mathop Q\limits^{.} }_{avg}}}}{{{{(\mathop m\limits^{.} Cp)}_{\hbox{min} }}({T_{h,i}} - {T_{c,i}})}}$$

The heat transfer per unit pumping power is calculated as

13 $${\mathop Q\limits^{.} }/P.P=\frac{{{{\mathop m\limits^{.} }_h}{c_h}\Delta {T_h}}}{{(\mathop m\limits^{.} \Delta {P_h}/{\rho _{_{h}}})+({{\mathop m\limits^{.} }_h}\Delta {P_c}/{\rho _c})}}$$

The thermal performance criteria can be calculates as^26^:

14 $$\eta ={\frac{Nu_c}{Nu_s}}\Bigg/ {\Bigg(\frac{f_c}{f_s}\Bigg)^{1/3}}$$

## Uncertainty analysis

The implication of the experimental error specifies the error of the measuring quantities. For the different calculated parameters, the uncertainty analysis was performed according to Holman^27^.

The error of the measured quantities to compute the uncertainty of several parameters such as *Re*,* h*,* Nu*,* f*, and *U*_*o*_. For the independent variables (*s*_*1*_, *s*_*2*_, *s*_*3*_, …, *s*_*n*_), considering the uncertainty in *W*_*1*_, *W*_*2*_, …, *W*_*n*_, and *W*_*R*_, the uncertainty in the experimental result was of the same order of magnitude, which can be given as follows;

15 $${W_R}=\sqrt {{{\left( {\frac{{\partial R}}{{\partial {s_1}}}{W_1}} \right)}^2}+{{\left( {\frac{{\partial R}}{{\partial {s_2}}}{W_2}} \right)}^2}+\,\,\cdots\,\,+{{\left( {\frac{{\partial R}}{{\partial {s_n}}}{W_n}} \right)}^2}}$$

For example, in order to determine *Nu*, it is important to consider the error of the measurement of such as *D*_*hy*_, convective heat transfer coefficient and *k*^28–31^. The measurement device uncertainty and accuracy are given in Table 2, the maximum error of *Nu* is no more than 4.6%.

In the current investigation the root sum square combination of the impact of each of individual inputs are examined to calculate the parameters;

16 $$\frac{{\partial Re}}{{Re}}=\sqrt {{{\left( {\frac{{\partial \mathop m\limits^{.} }}{{\mathop m\limits^{.} }}} \right)}^2}+{{\left( {\frac{{\partial {d_{hy}}}}{{{d_{hy}}}}} \right)}^2}+\,{{\left( {\frac{{\partial \mu }}{\mu }} \right)}^2}\,}$$

17 $$\frac{{\partial h}}{h}=\sqrt {{{\left( {\frac{{\partial \mathop Q\limits^{.} }}{{\mathop Q\limits^{.} }}} \right)}^2}+{{\left( {\frac{{\partial LMT{D_{avg}}}}{{LMT{D_{avg}}}}} \right)}^2}+{{\left( {\frac{{\partial A}}{A}} \right)}^2}}$$

18 $$\frac{{\partial Nu}}{{Nu}}=\sqrt {{{\left( {\frac{{\partial h}}{h}} \right)}^2}+{{\left( {\frac{{\partial {d_{hy}}}}{{{d_{hy}}}}} \right)}^2}+{{\left( {\frac{{\partial k}}{k}} \right)}^2}}$$

19 $$\frac{{\partial f}}{f}=\sqrt {{{\left( {\frac{{\partial \Delta P}}{{\Delta P}}} \right)}^2}+{{\left( {\frac{{\partial L}}{L}} \right)}^2}+{{\left( {\frac{{\partial {d_{hy}}}}{{{d_{hy}}}}} \right)}^2}+{{\left( {\frac{{\partial \mathop m\limits^{.} }}{{\mathop m\limits^{.} }}} \right)}^2}}$$

20 $$\frac{{\partial \varepsilon }}{\varepsilon }=\sqrt {{{\left( {\frac{{\partial \mathop Q\limits^{.} }}{{\mathop Q\limits^{.} }}} \right)}^2}+{{\left( {\frac{{\partial \mathop {{Q_{Max}}}\limits^{.} }}{{\mathop {{Q_{Max}}}\limits^{.} }}} \right)}^2}}$$

21 $$\frac{{\partial \left( {{{\mathop Q\limits^{.} } \mathord{\left/ {\vphantom {{\mathop Q\limits^{.} } {PP}}} \right. \kern-0pt} {PP}}} \right)}}{{\left( {{{\mathop Q\limits^{.} } \mathord{\left/ {\vphantom {{\mathop Q\limits^{.} } {PP}}} \right. \kern-0pt} {PP}}} \right)}}=\sqrt {{{\left( {\frac{{\partial \mathop Q\limits^{.} }}{{\mathop Q\limits^{.} }}} \right)}^2}+{{\left( {\frac{{\partial \mathop m\limits^{.} }}{{\mathop m\limits^{.} }}} \right)}^2}+{{\left( {\frac{{\partial \Delta P}}{{\Delta P}}} \right)}^2}}$$

**Table 2:** The range, accuracy and uncertainties of measuring devices. .

Instruments	Range	Accuracy (%)	Uncertainty (%)
Rotameter, kg.s^−1^	0.016–0.3	± 0.5	± 0.17
Thermocouple K-type, °C	−200–1200	± 0.1	± 0.16
Digital differential pressure transmitter, kPa	0.5–500	± 0.5	± 01.16
Reynolds number	5000–50,000	-	± 2.8
Friction factor	-	-	± 4.6
Nusselt number	-	-	± 4.6
Effectiveness	-	-	± 5.4
Heat transfer per pumping power	-	-	± 5.8

## Results and discussion

The set of experiments involves one smooth tube and five twisted spiral tubes at various twisted spiral pitch ratios and twisted spiral depths. Preliminary tests are first conducted on a smooth tube. Second, experiments are conducted on twisted spiral tubes with different pitches and different depths. The thermofluid performance criterion of the heat exchanger with a double-twisted spiral tube was presented as follows: the Nusselt number, overall heat transfer coefficient, friction factor and pressure drop. This criterion was demonstrated at different pitch ratios, depth ratios, and flow configurations.

### Influence of flow arrangements

**Fig. 3:**
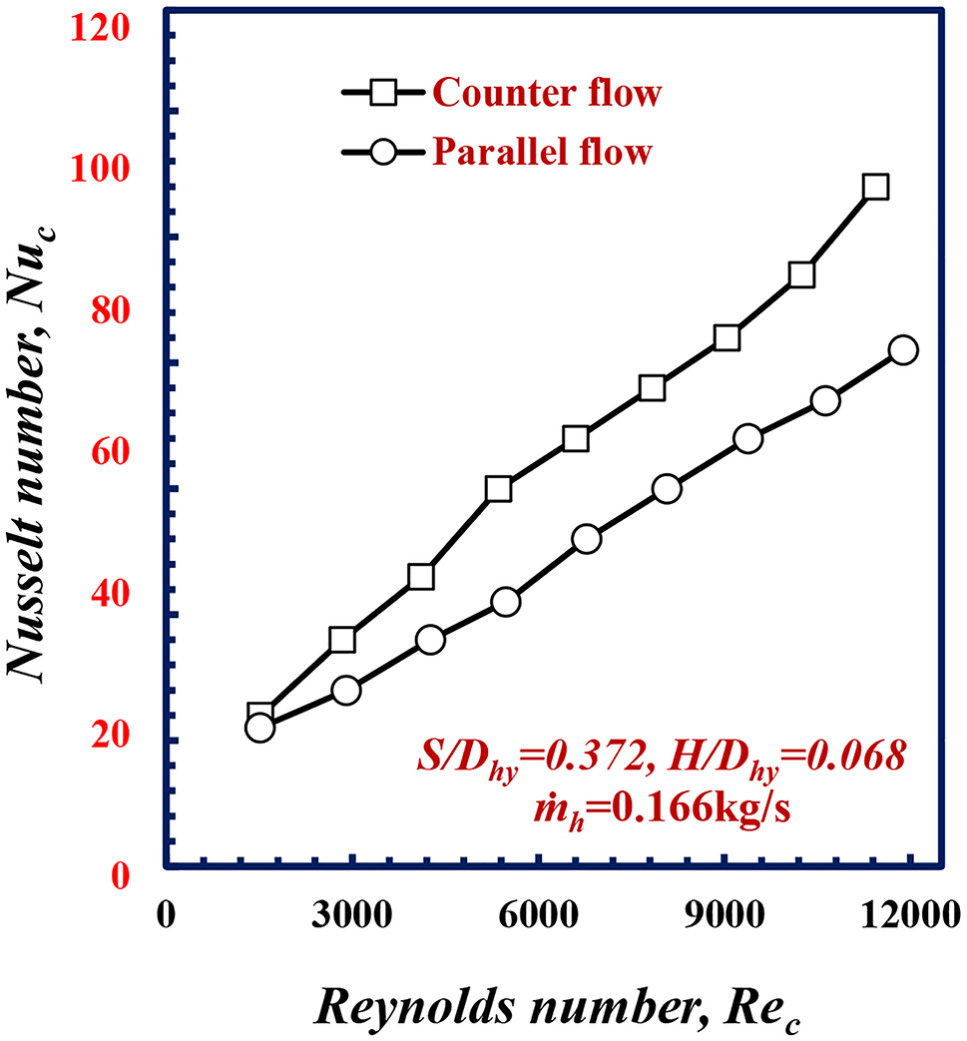
Variation of Nusselt Number with Reynolds Number of twisted spiral tube at *S/D*_*hy*_= 0.372 and *H/D*_*hy*_= 0.068

The effects of the parallel and counter flow arrangements on the thermofluid characteristics were shown for the test spacimen No. 2 as a test sample in which has a twisted spiral pitch of 5.2 mm and a twisted spiral depth of 0.95 mm (corresponding to *S/D*_*hy*_ = 0.372 and *H/D*_*hy*_= 0.068) and hot water flow rate of 10 lit. /m. *Nu* against *Re*, are illustrated in Fig. 3. These figure showed that, the Nusselt number *Nu* increased with *Re.* this is due to increasing in fluid velocity and the turbulence level in which enhance the convective heat transfer coefficient. For the same cold water *Re*_*c*_ of 9000 and the Nusselt number *Nu* for counter flow were higher than those for parallel flow by 16%. This can be attributed to an increase in the water temperature difference *T*_*LMTD*_ between the cold water in annulus and the hot water in the inner corrugated tube in the counter flow pattern, compared to parallel flow pattern. This increase leads to an increase in the heat transfer rate and, consequently, an increase in *Nu*.

**Fig. 4:**
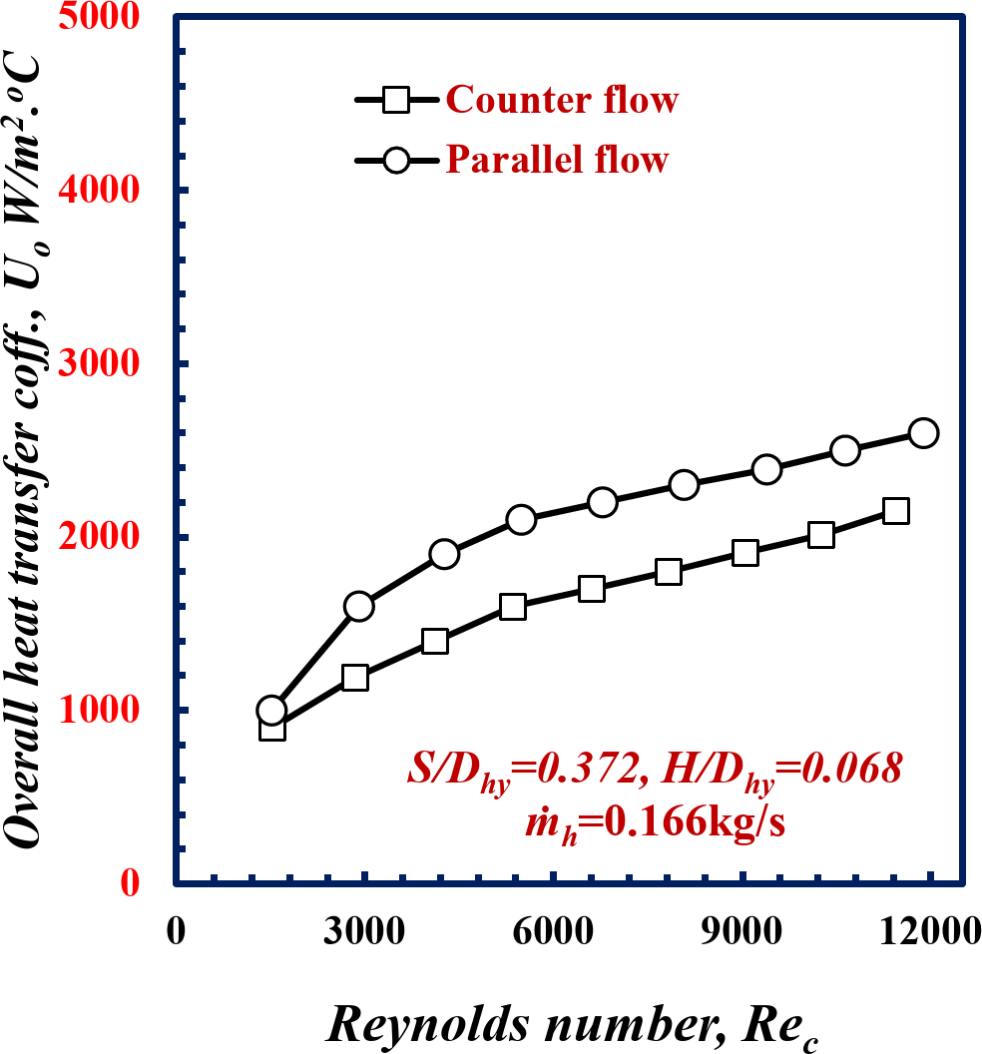
Variation of overall heat transfer coefficient with Reynolds Number of twisted spiral tube at *S/D*_*hy*_ = 0.372 and *H/D*_*hy*_ = 0.068

The effects of the parallel and counter flow arrangements on the overall heat transfer coefficient versus *Re* are illustrated in Fig. 4. At the same cold water Reynolds number of 9000, the overall heat transfer coefficient for counter flow was 8.7% greater than that for parallel flow. Through the counter flow the temperature differences between the hot and cold fluids is approximately constant along the heat exchanger length, this lead to increase the heat transfer rates for the same surface area. So, the outlet temperature of the hot fluid in the counter-flow can be cooked even less than that of cold fluid which cannot be done in case of a parallel flow case.

**Fig. 5:**
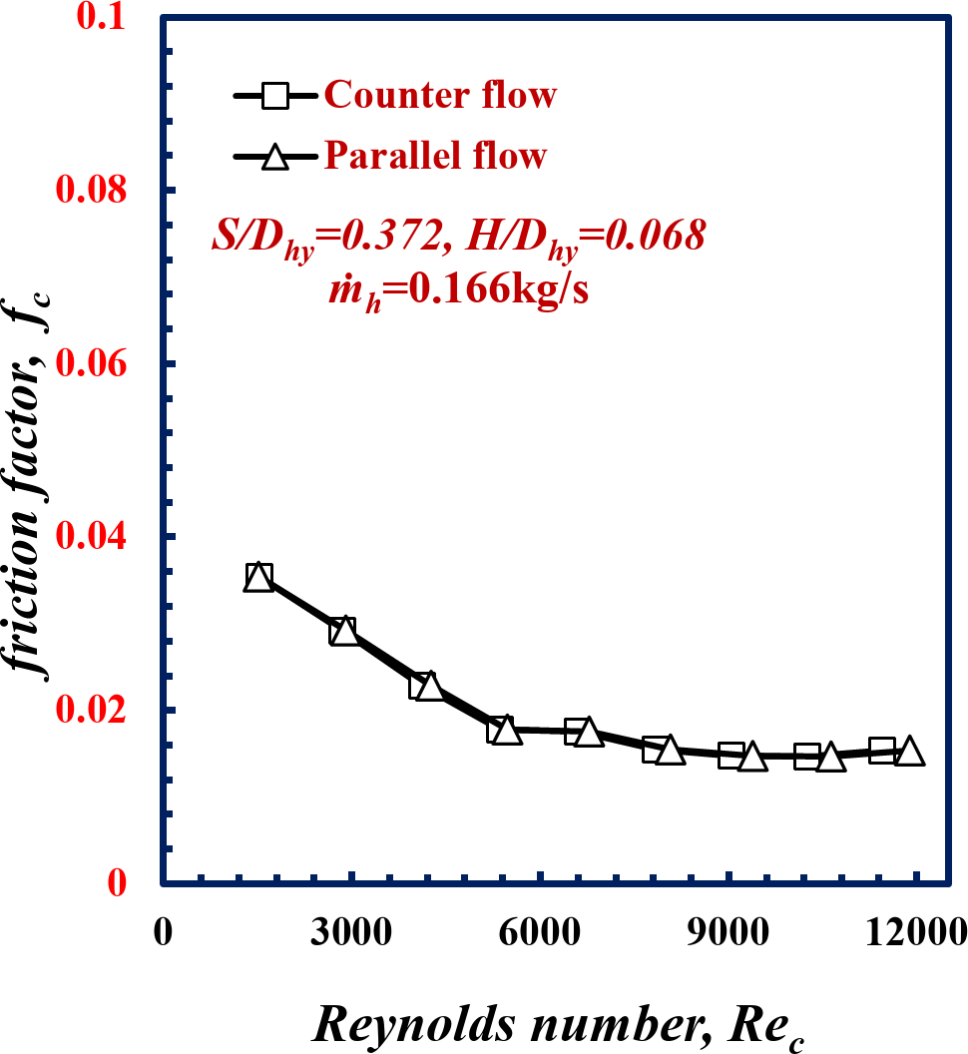
Variation of friction factor with Reynolds Number of twisted spiral tube at *S/D*_*hy*_=0.372 and *H/D*_*hy*_=0.068

The friction factor *f* versus the Reynolds number is shown in Fig. 5. Notably, the friction factor *f* decreases with *Re*. with increasing of *Re* the momentum force, the mixing fluid flow due to spiral twisted tube increased, and amplifying turbulence flow in which decreasing the viscous boundary layer thickness (viscous force) occurred and hence the decreasing in *f* occurred. For the *Re*_*c*_ number of 9000, the friction factor *f*_*c*_ for counter flow was the same value as that for parallel flow.

**Fig. 6:**
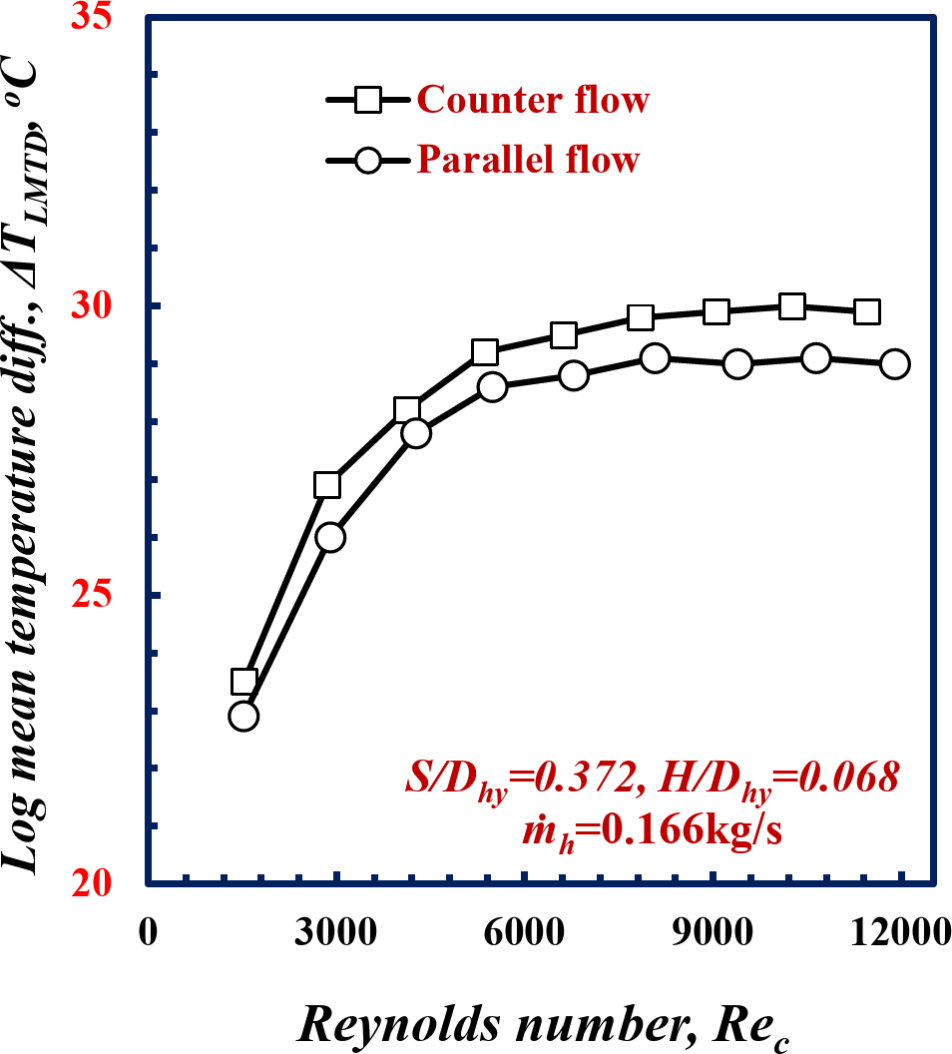
Variation of *∆T*_*LMTD*_ with Reynolds Number of twisted spiral tube at *S/D*_*hy*_=0.372 and *H/D*_*hy*_=0.068

The logarithmic mean temperature difference *∆T*_*LMTD*_ versus the Reynolds number, *Re*, for a pitch of 5.2 mm is illustrated in Fig. 6. Notably, the logarithmic mean temperature difference increases with *Re*. For the *Re*_*c*_ number of 9000, the *∆T*_*LMTD*_ for counter flow was higher than that of parallel flow by 6.7%. For the counter flow pattern, the cold fluid enters the heat exchanger at the exit of the hot fluid, which allows for the exchange more heat between the hot and cold fluids. For this reason, the change in the temperature difference is less than in the parallel flow.

### **Influence of twisted spiral pitch ratios**

**Fig. 7:**
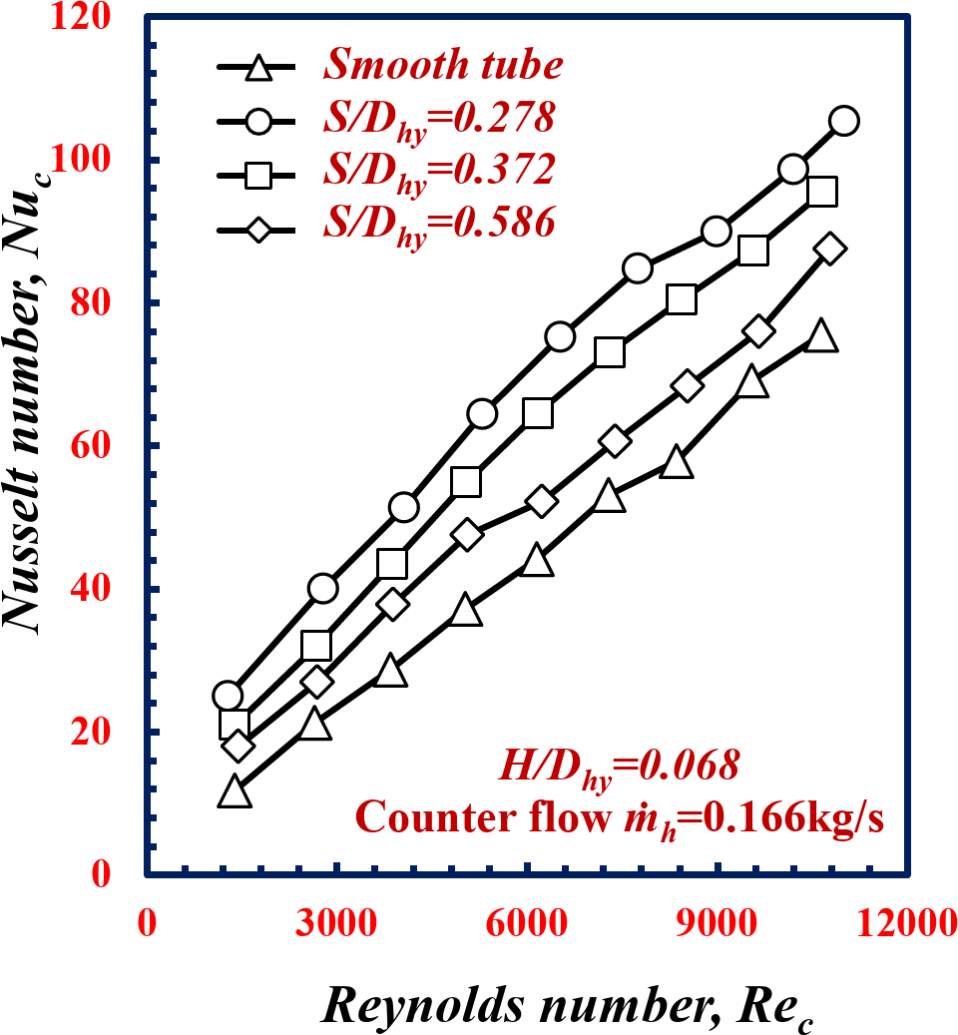
Variation of Nusselt Number with Reynolds Number of twisted spiral tube at different twisted spiral pitch ratios

The impact of twisted spiral pitch ratios is a point of concern. The results are showed at different twisted spiral pitches ratios, *S/D*_*hy*_, of 0.278, 0.372 and 0.586 corresponding to *(S)* of 3.9, 5.2, and 8.2 mm and constant depth *(H)* as well as hot water mass flow rate of 0.166 kg/s. Figure 7. Illustrates relationship between the Nusselt number of cold water and the Reynolds number of the smooth tube and various twisted spiral tube. From Fig. 7 it is apparent that the Nusselt number *Nu* increases with decreasing *(S/D).*Notably, at *Re* = 9000, *Nu* increases by 38%, 30.1%, and 11.6% at *S/D*_*hy*_ of 0.278, 0.372 and 0.586, respectively, in comparison with the results obtained for the smooth tube under the same conditions. This means that the Nusselt number *Nu* is inversely proportional to *S/D*_*hy*_ at constant *H*. Decreasing the twisted spiral pitch ratio, *S/D*_*hy*_, this leads induce axial flow by swirling the fluid, improved mixing intensity of fluid flow, disrupt the boundary layer thickness and the flow becomes more turbulent. This increases water temperature difference Δ*T*_*LMTD*_ between the annulus cold water and the hot water, and leads to an increase in the heat transfer rate and, consequently, an increase in *Nu*.

**Fig. 8:**
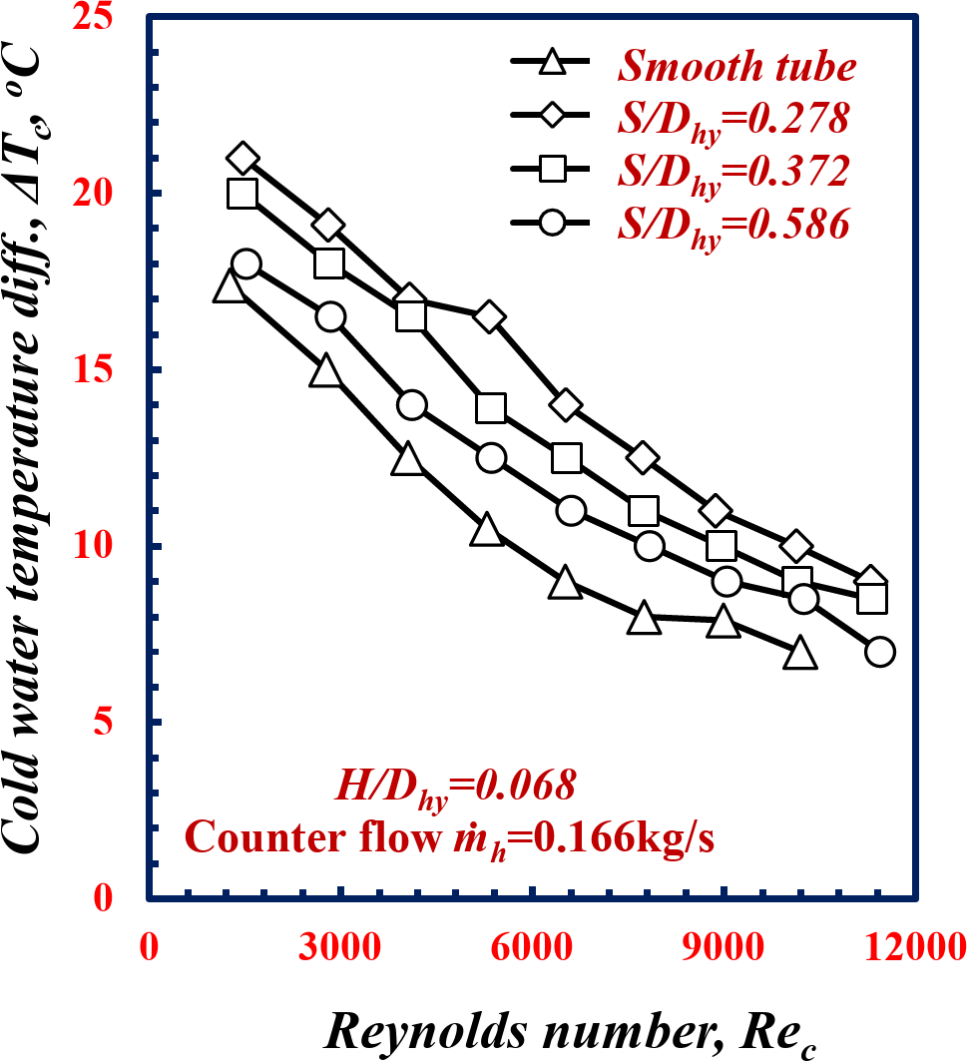
Variation of Variation of *∆T*_*c*_ with Reynolds number of twisted spiral tube at different twisted spiral pitch ratios.

**Fig. 9:**
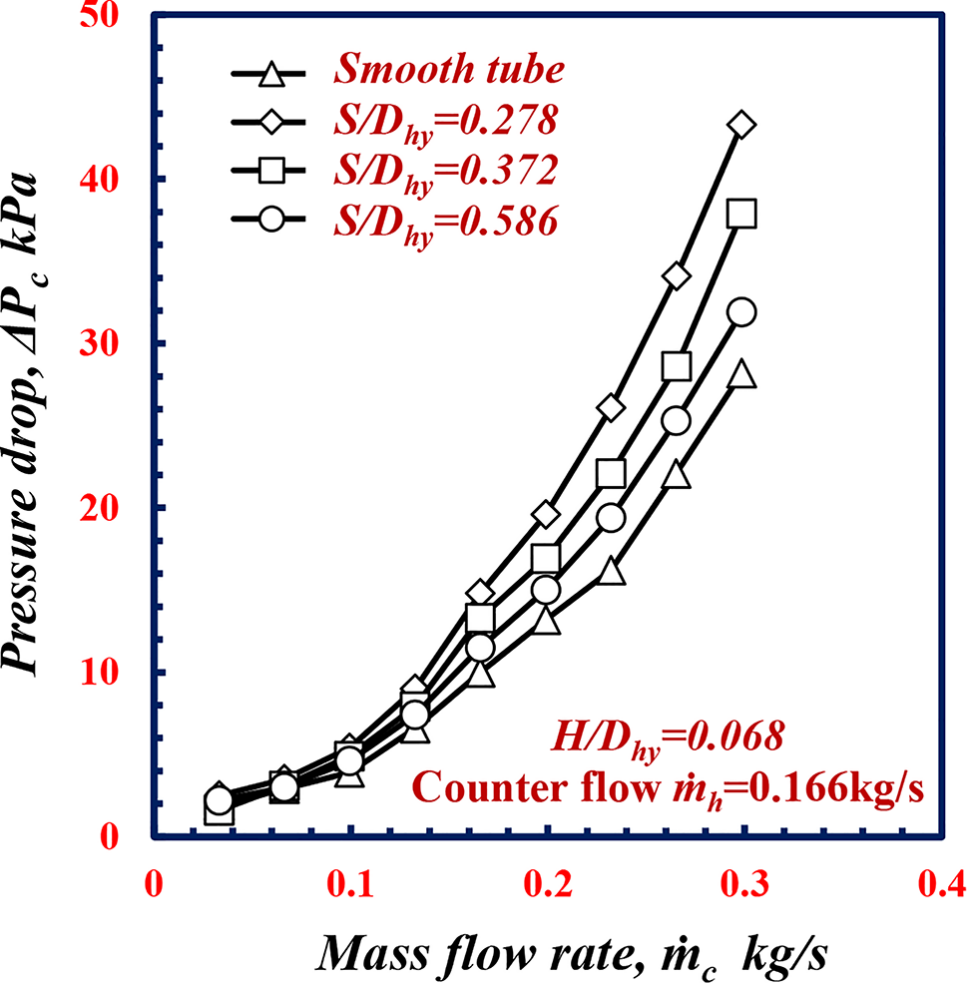
Variation of pressure drop with mass flow rate of twisted spiral tube at different twisted spiral pitch ratios.

Figure 8 shows the variation in ∆*T*_*c*_ versus *Re*_*c*_ for various twisted spiral pitches of twisted tube *S /D*_*hy*_. Figure 9 shows the decreasing in ∆*T*_*c*_ for all the geometries of *S/D*_*hy*_ with increasing *Re*_*c*_. For the same *Re*_*c*_ of 9000, the ∆*T*_*c*_ values of the twisted spiral tube with *S/D*_*hy*_ of 0.278, 0.372 and 0.586, are greater than the ∆*T*_*c*_ values for the smooth tube by 34.6%, 26.6% and 14.1%, respectively. Decreasing the twisted spiral pitch ratio, *S/D*_*hy*_, has a significant effect on the mixing of the fluid in the boundary layer and increases the turbulent intensity of the fluid flow, which leads to an increase in the heat transfer rate.

**Fig. 10:**
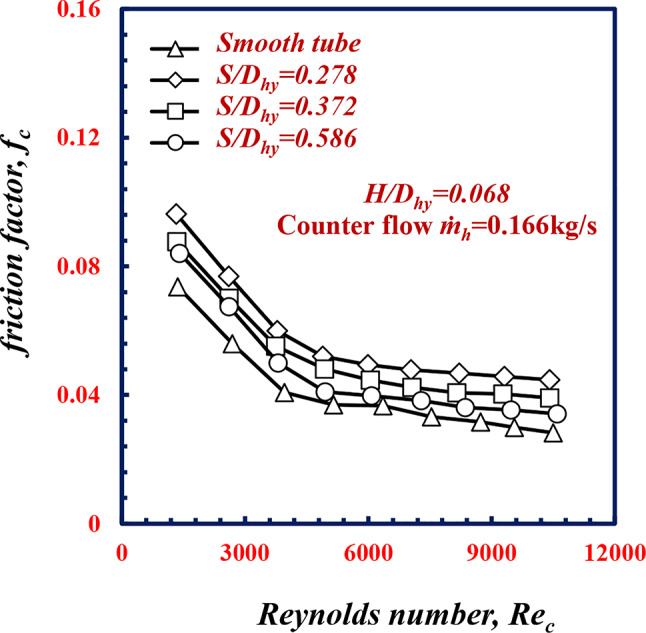
Variation of friction factor with Reynolds Number of twisted spiral tube at different twisted spiral pitch ratios.

Figure 9 Illustrates the relationship of the pressure drop *ΔP* for cold water with the *m*_*c*_ of the smooth tube and twisted spiral tube at different *S/D*_*hy*_ of 0.278, 0.372 and 0.586. From this figure, it is apparent that the pressure drop *ΔP* increases with decreasing twisted spiral pitch ratio *S/D*_*hy.*_ Notably, at *m*_*c*_ *=*0.23 kg/s, the pressure drop *ΔP* increases by 61.1%, 36.4%, and 19.7% at *S/D*_*hy*_ of 0.278, 0.372 and 0.586, respectively, which is greater than the results obtained for the smooth tube under the same conditions. Owing to flow swirls that induced due to the twisted spiral pitch increasing along the surfaces of the tube, which causes the fluid more turbulent, the turbulent flow leads to an increase *f* in which increases the pressure drop. Figure 10 shows the variation in the friction factor *f*_*c*_ versus *Re*_*c*_ for various *S/D*_*hy.*_ Figure 10 shows that *f* decreases for all the geometries of *S/D*_*hy*_ with increasing *Re*_*c*_. For the same *Re*_*c*_ of 9000, the friction factors of the twisted spiral tube pitches ratios of *S/D*_*hy*_ of 0.278, 0.372 and 0.586, are 33.2%, 21.8% and 9.1% greater than the *f*_*c*_ value for the smooth tube, respectively. While the twisted spiral pitch ratio decreases along the surface of the tube, this causes more turbulence in the flow However, this enhancement in heat transfer comes with a trade-off of a higher pressure drop.

**Fig. 11:**
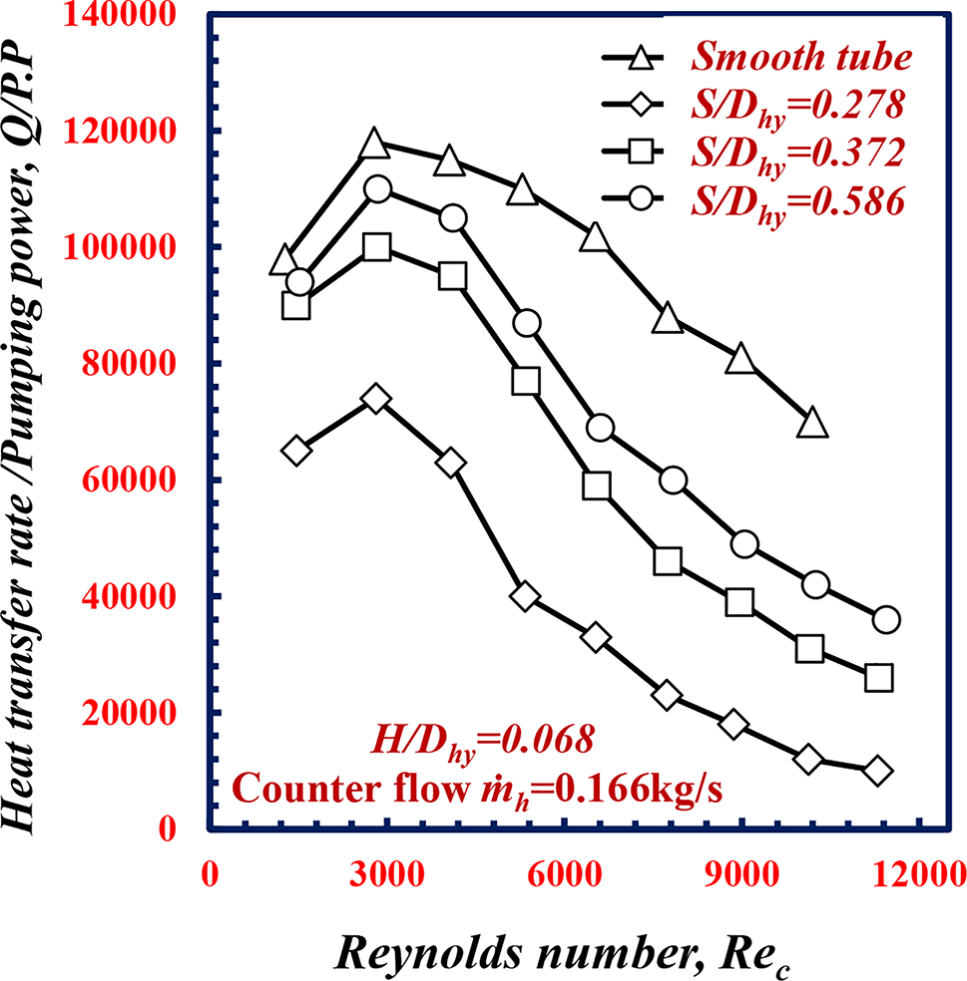
Variation of *Q/P.P* with Reynolds number of twisted spiral tube at different twisted spiral pitch ratios

Figure 11 shows the variation in *Q/P.P* versus *Re*_*c*_ for various pitches of twisted spiral *S/D*_*hy*_. Figure 11 shows that the *Q/P.P* decreases for all the geometries of *S/D*_*hy*_ with increasing *Re*_*c*_. For the same *Re*_*c*_ of 9000, the *Q/P.P* values of the *S/D*_*hy*_ of 0.278, 0.372 and 0.586, are less than the values of *Q/P.P* values for the smooth tube were 34.6%, 26.6% and 14.1%, respectively. Increasing the heat transfer rate due to *S/D*_*hy*_ comes with a trade-off of a higher pressure drop in which a main factor of *P.P* (volumetric flow rate multiplied with pressure drop). So the increase in heat transfer rate is lower than the increase in *P.P*, this demonstrate why the smooth test specimen present higher *Q/P.P.*

**Fig. 12:**
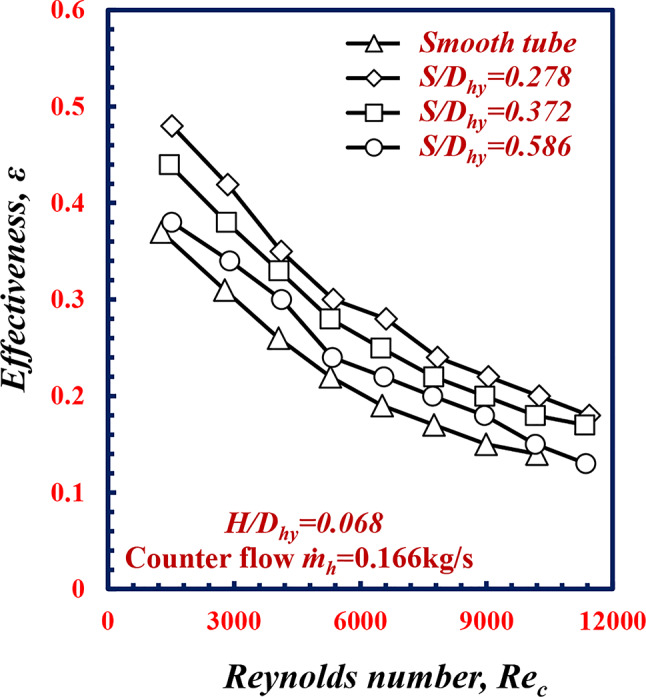
Variation of effectiveness *ε* with Reynolds number of twisted spiral tube at different twisted spiral pitch ratios

Figure 12 shows the relationship between the effectiveness *ε* and the Reynolds numbers of the smooth tube and twisted spiral tubes at different *S/D*_*hy*_. From this figure, it is apparent that the effectiveness *ε* decreases with increasing pitch *S/D*_*hy*_. Notably, at *Re* = 9000, *ε* increases by 37.97%, 20.25%, and 13.92% at *S/D*_*hy*_ of 0.278, 0.372 and 0.586, respectively, in comparison with the results obtained in the case of the smooth tube under the same conditions, owing to the decrease the twisted spiral pitch ratio *S/D*_*hy*,_ this leads increase the swirling flow and mixing intensity of fluid flow, This lead to increase water temperature difference Δ*T* between the annulus cold water and the hot water, in which leads to increasing in the heat transfer rate compared to the maximum heat transfer rate and hence an increase in the effectiveness occurred.

### Influence of the twisted spiral depth

**Fig. 13:**
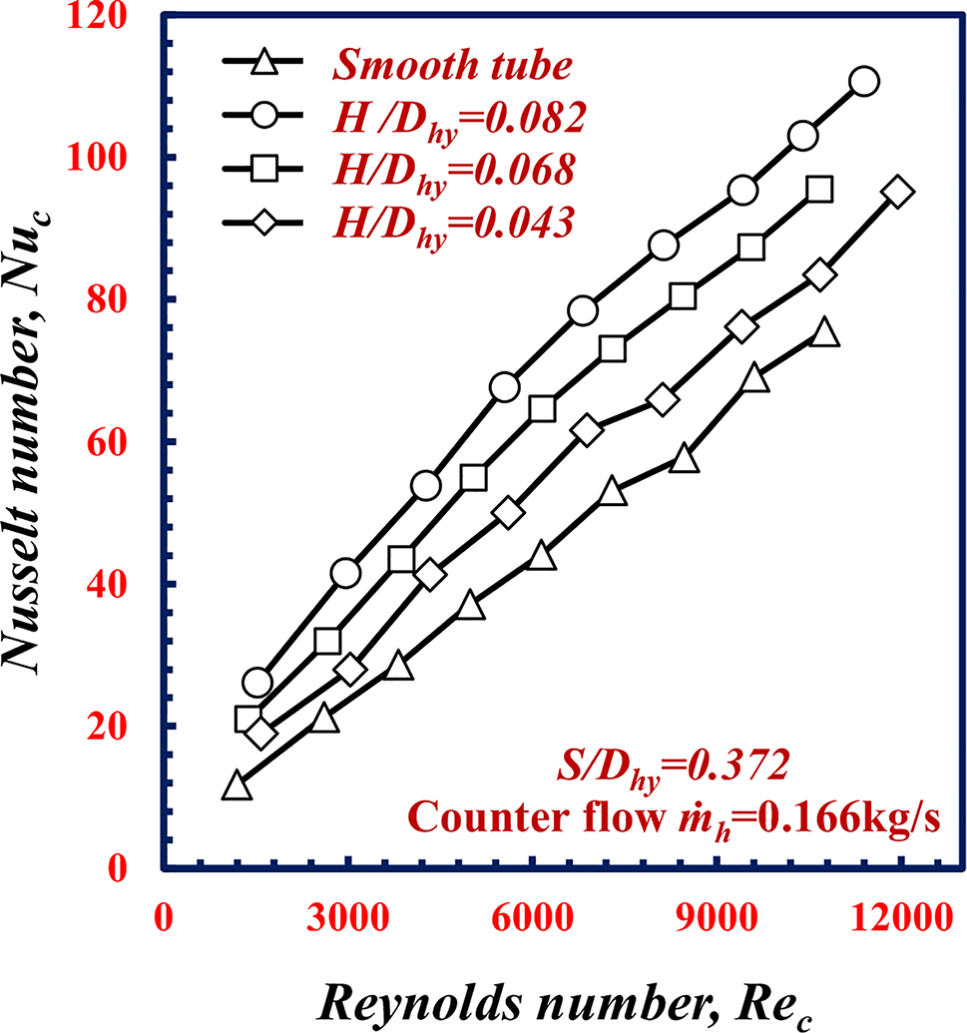
Variation of Nusselt number with Reynolds Number of twisted spiral tube at different twisted spiral depth ratios.

The influence of the twisted spiral depth ratios *H/D*_*hy*_ is a point of interest. Three different *H/D*_*hy*_ of 0.043, 0.068 and 0.082 (corresponding to *H* of 0.6, 0.95, and 1.15 mm) is examined at constant *S/D*_*hy*_ of 0.372 and constant hot water mass flow rate of 0.166 kg/s. Figure 13 shows the relationship between the Nusselt number Nu of cold water and the Re values of the smooth tube and twisted spiral tube at *H/D*_*hy*_. From this figure, it is appare that *Nu*_*c*_ increases with increasing *H/D*_*hy*_ for all cases. Notably, at *Re* = 9000, *Nu*_*c*_ increases by 44.9%, 30.1%, and 14.7% at *H/D*_*hy*_, of 0.043, 0.068 and 0.082 respectively, in comparison with the results obtained for the smooth tube under the same conditions. Additionally, the *Nu* increases with increasing Reynolds number for all the cases. As the twisted spiral depth increases, the turbulent flow increases because the twisted spiral corrugation increases, which enhancing the fluid mixing and the turbulence flow intensity in which leads to decrease the boundary layer thickness and improves the water temperature difference (T_*LMTD)*_ between the hot water and the cold water in annulus. This increase leads to an increase in the heat transfer rate and consequently an increase in *Nu*_c_ occurred.

**Fig. 14:**
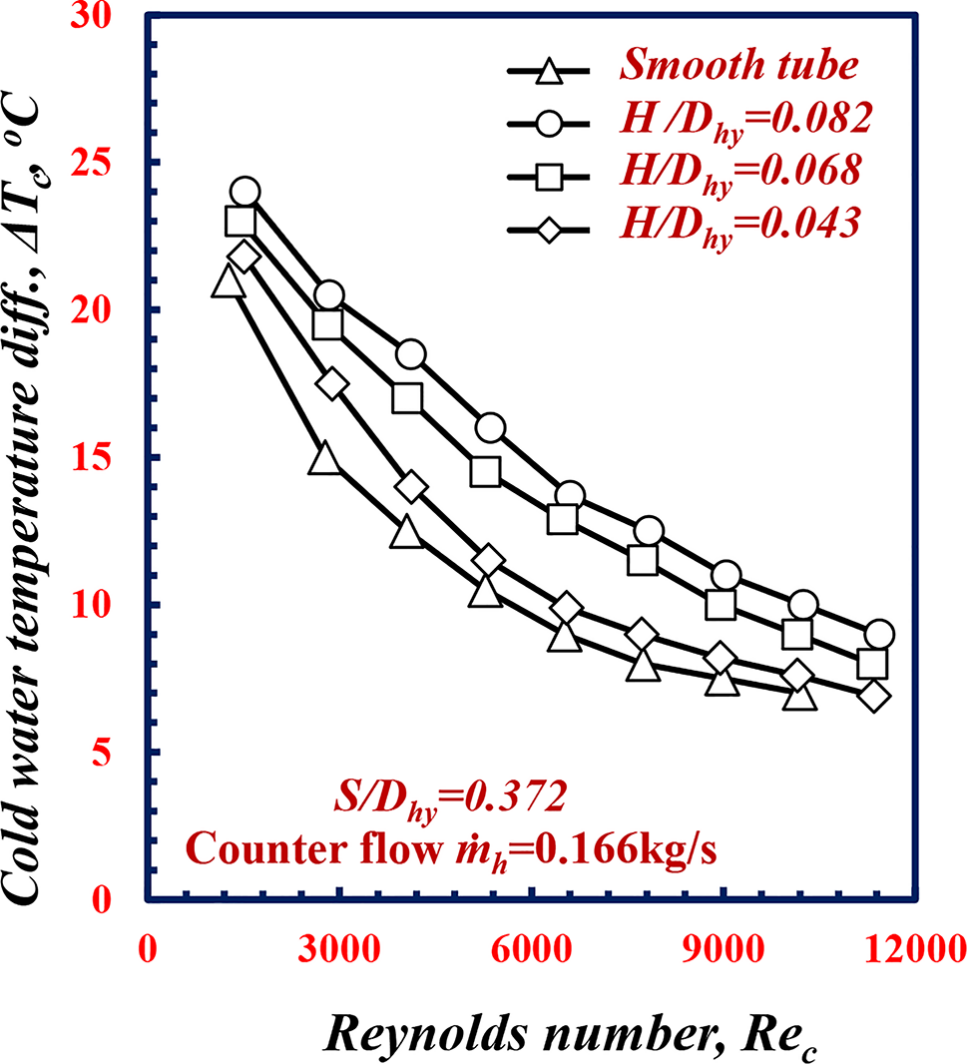
Variation of ∆*T*_c_ with Reynolds number of twisted spiral tube at differenttwisted spiral depth ratios﻿

Figure 14 shows the variation in ∆*T*_*c*_ versus *Re*_*c*_ for various twisted spiral tube depth ratios, *H/D*_*hy*_. Figure 14 shows that ∆*T*_*c*_ decreases for all the geometries of *H/D*_*hy*_ with increasing *Re*_*c*_ for all cases and all the twisted tubes present a larger values of ∆*T*_*c*_ compared to the smooth tube. For the same *Re*_*c*_ of 9000, the ∆*T*_*c*_ values of the *H/D*_*hy*_ of 0.043, 0.068 and 0.082 are greater than the values of ∆*T*_*c*_ for the smooth tube by 13.3%, 33.33% and 48%, respectively. This is because the effect of twisted spiral depth has a significant effect on fluid turbulence. As the depth of the twisted spiral tube increases, this increase leads to an increase in the fluid flow resistance, which increases the heat transfer rates.

**Fig. 15:**
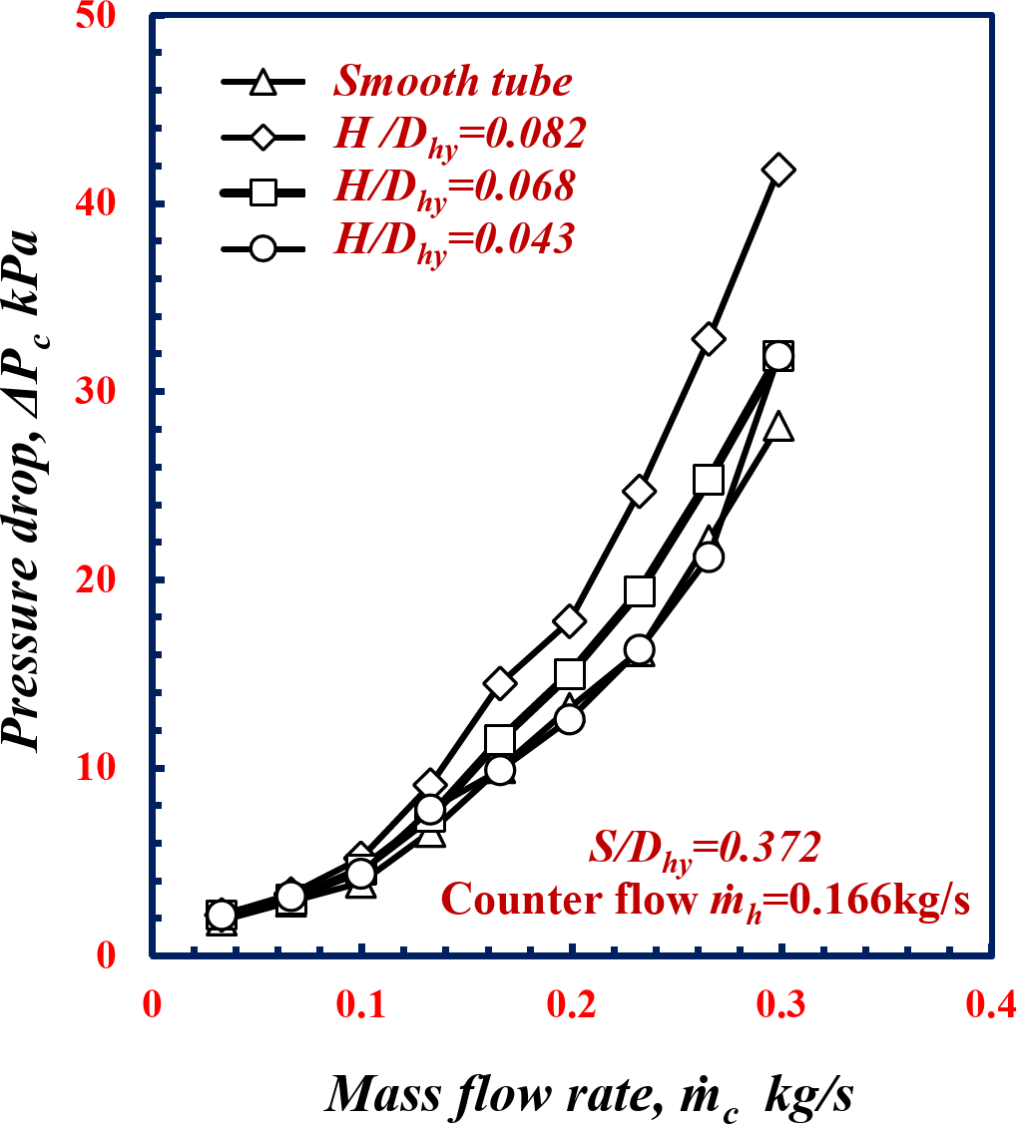
Variation of pressure drop, with mass flow rate of twisted spiral tube at different twisted spiral depth ratios﻿

Figure 15 shows the relationship between the pressure drop *ΔP*_*c*_ and the ${\mathop m\limits^{.}}_c$ of the smooth tube and twisted spiral tube at different *H/D*_*hy*_. From this figure, it is apparent that *ΔP*_*c*_ increases with increasing *H/D*_*hy*_. Notably, at ${\mathop m\limits^{.}}_c$ of 0.23 kg/s, the pressure drop *ΔP*_*c*_ increases by 52.4%, 19.7%, and 0.6% at *H/D*_*hy*_ of 0.082, 0.068 and 0.043, respectively, in comparison with the results obtained for the smooth tube under the same conditions. Owing to the increase in the depth, when the *H/D*_*hy*_ of the twisted spiral tube increases, the flow resistance increases along the surface of the tube, which causes more turbulent flow and fluid mixing, this lead to decreasing the boundary layer and enhance the thermal characteristics at the expense of increasing *f* and consequently increasing the pressure drop occurred.

**Fig. 16:**
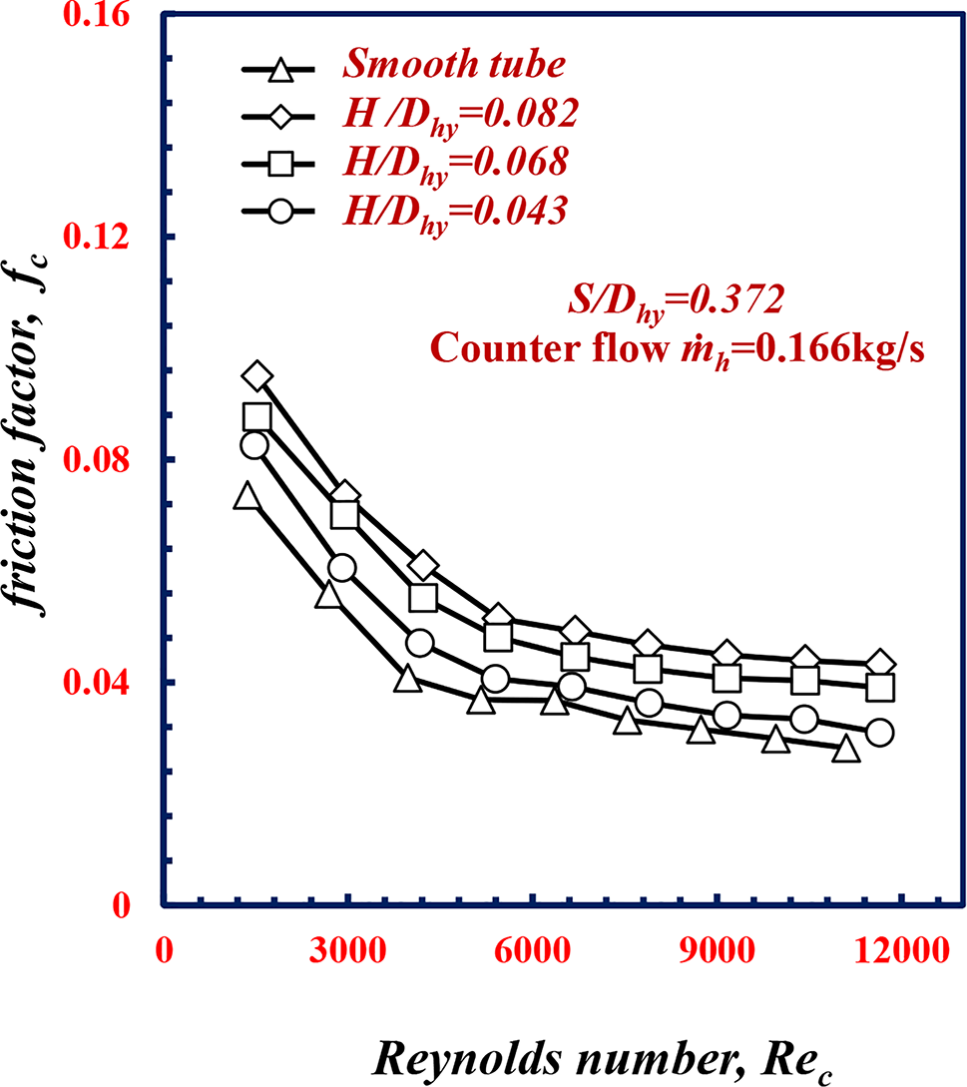
Variation of friction factor with Reynolds Number of twisted spiral tube at different twisted spiral depth ratios ﻿

Figure 16 shows the variation in the friction factor *f*_*c*_ versus *Re*_*c*_ for various *H/D*_*hy*_. Figure 16 shows that the friction factor *f*_*c*_ decreases for all geometries of *H/D*_*hy*_ with increasing *Re*_*c*_ for all cases. For the same *Re*_*c*_ of 9000, the *f*_*c*_ at *H/D*_*hy*_ of 0.082, 0.068 and 0.043, is 36.4%, 21.8% and 6.1% greater than the smooth tube value, respectively. Owing to twisted spiral tube depth, when the *H/D*_*hy*_ increases, the axial swirls of flow increasing along the surfaces of the tube, this causes more turbulence in the flow, which leads to an increase in the friction factor and pressure drop. Friction loss can be attributed to the blockage of flow passages, long residence times or flow paths, and vortex flows, which results in higher local velocities, larger pressure drops and greater heat transfer rate.

**Fig. 17:**
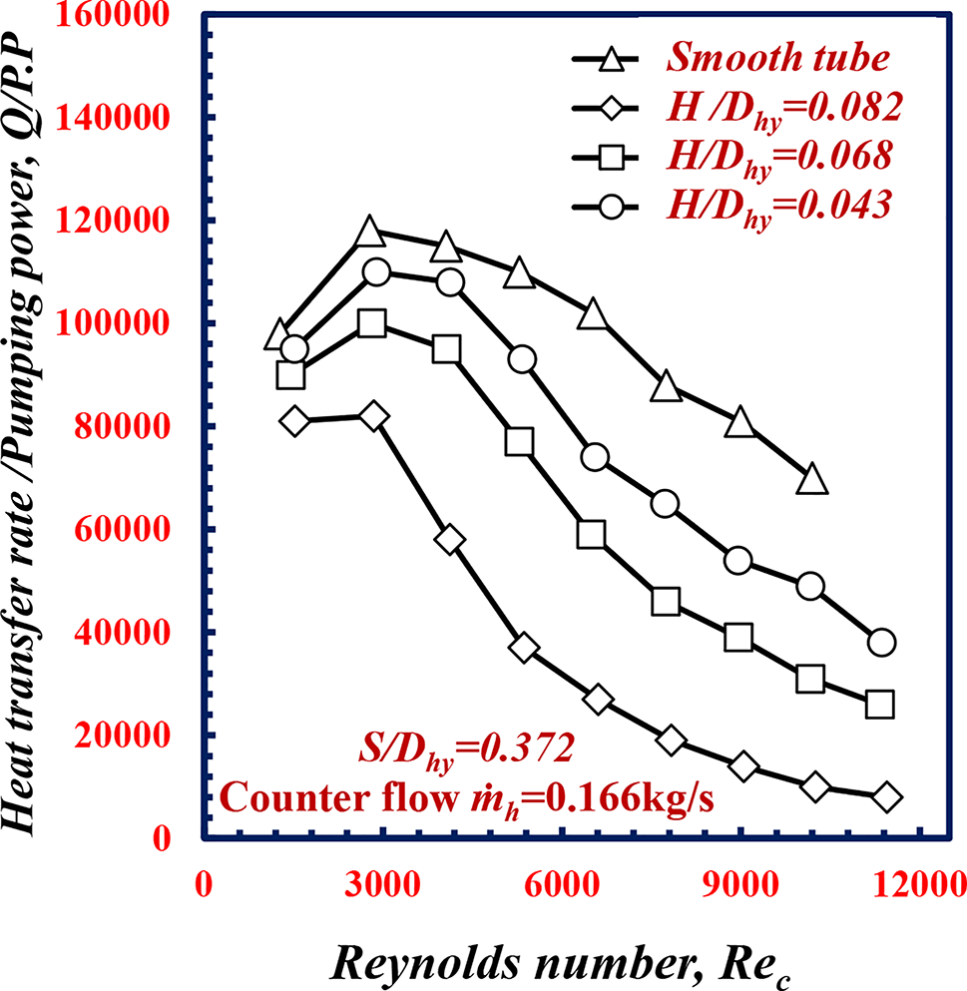
Variation of *Q/P.P*, with Reynolds number of twisted spiral tube at different twisted spiral depth ratios﻿

Figure 17 shows the variation in *Q/P.P* versus *Re*_*c*_. it is clearly to notice that the *Q/P.P* decreases for all the geometries with increasing *Re*_*c*_, and all of them are less than that of the smooth tube. For the same *Re*_*c*_ of 9000, the *Q/P.P* values of the twisted spiral tube depths ratios, *H/D*_*hy*_ of 0.043, 0.068 and 0.082 are less than the values of *Q/P.P* values for the smooth tube. This is because the increase in *P.P* in twisted spiral tubes compared to the smooth tubes. So the increase in heat transfer rate is lower than the increase in *P.P*, this demonstrate why the smooth test specimen present higher *Q/P.P.*

**Fig. 18:**
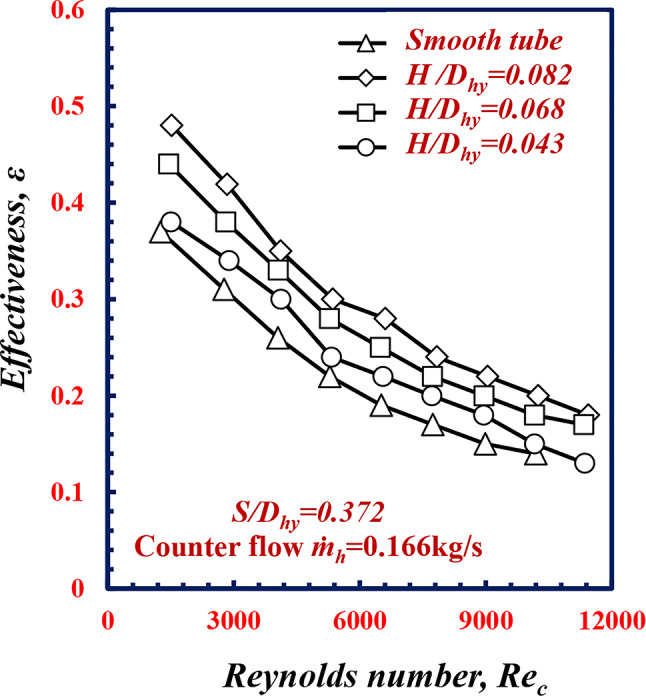
Variation of effectiveness *ε*, with Reynolds number of twisted spiral tube at different twisted spiral depth ratios﻿

Figure 18 shows the relationship between the effectiveness *ε* and the Reynolds number of the smooth tube and twisted spiral tube at different *H/D*_*h*__y_. From the figure, it is appare that the *ε* increases with increasing *H/D*_*hy*_. Notably, at *Re*_*c*_ =9000, *ε* increases by 12.65%, 20.25%, and 32.90% at *H/D*_*hy*_, of 0.043, 0.068 and 0.082, respectively, in comparison with the results obtained in the case of the smooth tube under the same conditions. Due to an increase in twisted spiral pitch, *H*, the fluid turbulence of fluid flow, mixing and swirl flow across the axial length of the tube increased and the increase in the hot water temperature difference occurred. this leads to an increase in the heat transfer rate (*Q*_*avg*_) relative to the maximum heat transfer rate (*Q*_*max.*_) and hence an increase in the effectiveness ocurred.

### The thermal performance criteria

**Fig. 19:**
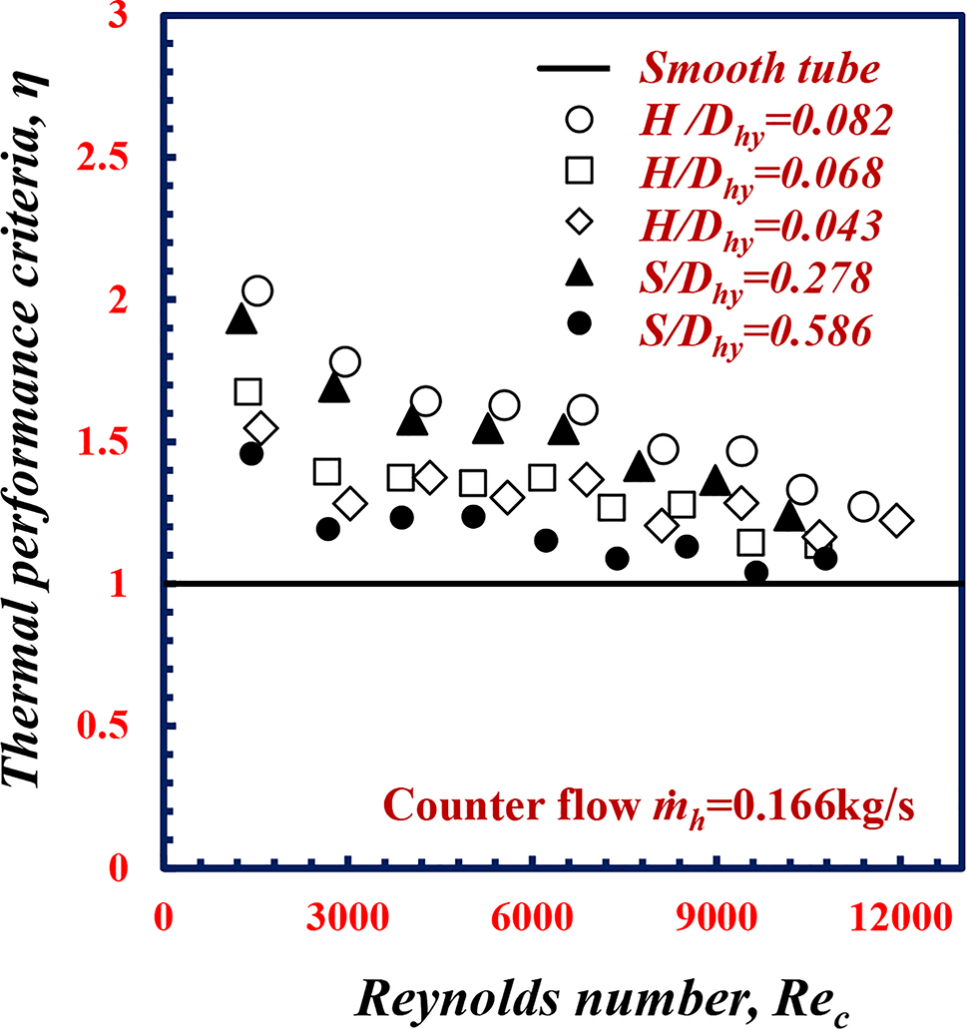
Variation of thermal performance criteria, with Reynolds number of twisted spiral tubes.

The thermal performance criteria, *η*, is a factor that define the values of enhancement that occurred during involving the twisted spiral tube as a heat exchanger. The thermal performance criteria is defined according to Eq. 14. Figure 19 shows *η* against *Re*_*c*_ at different *S/D*_*hy*_ and *H/D*_*hy*_ values It can be concluded that when *Re*_*c*_ increases, *η* decreases. In addition, the maximum *η* value reached 1.93 and 2.03 for test specimens 1 and 5, respectively.

### Validation of twisted spiral tubes

**Fig. 20:**
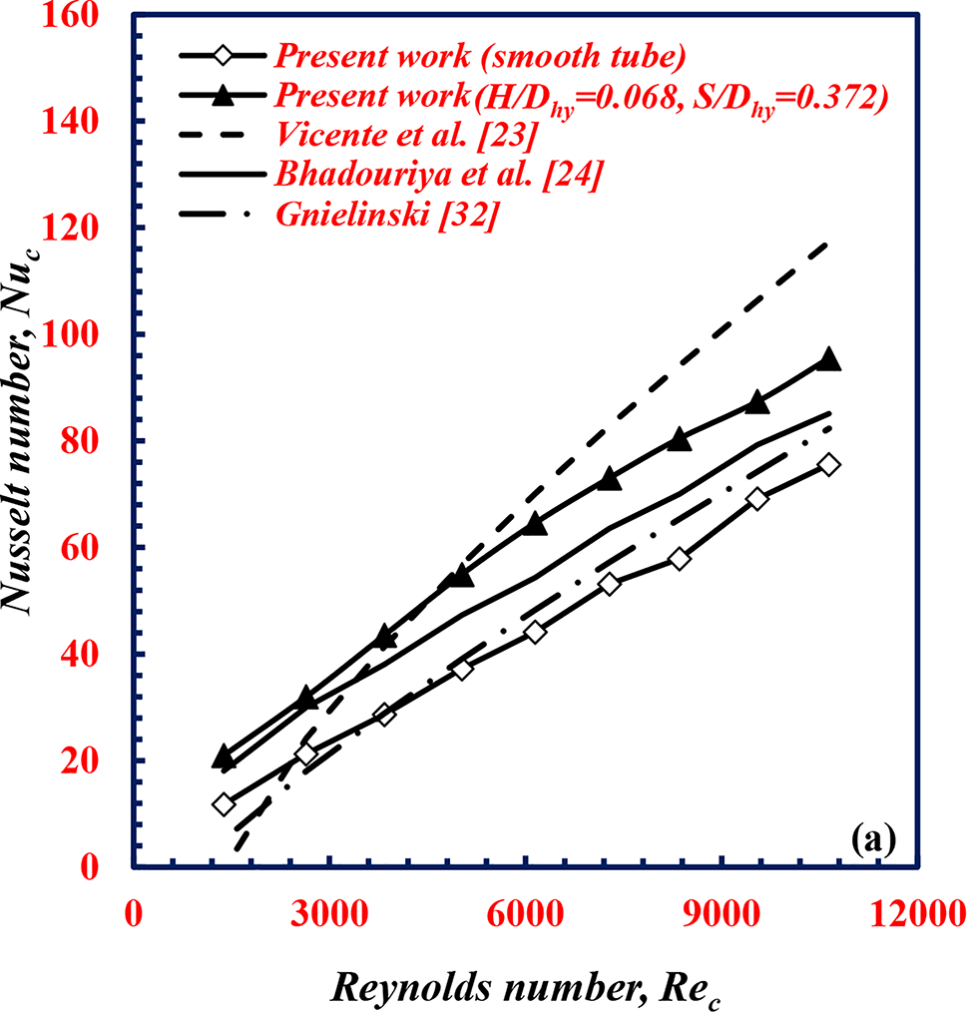
Validation of *Nu*_*c*_ with *Re*_*c*_ of twisted spiral tube.

The experimental facility is validated by comparing the *Nu* value of the cold water with the published correlations (Table 3) of Vicente et al.^23^, Bhadouriya et al.^24^ for twisted tubes, and Gnielinski^32^ for smooth tube. As depicted in Fig. 20. The figure shows that the present data for the twisted corrugated tube agree well with the correlations of Vicente et al.^23^, and Bhadouriya et al.^24^ with average deviations of 16.9%, and − 12.9%, respectively, whereas the present results for the smooth tube presented lower values than the Gnielinski Eq. with a variation of 12.9%.

**Fig. 21:**
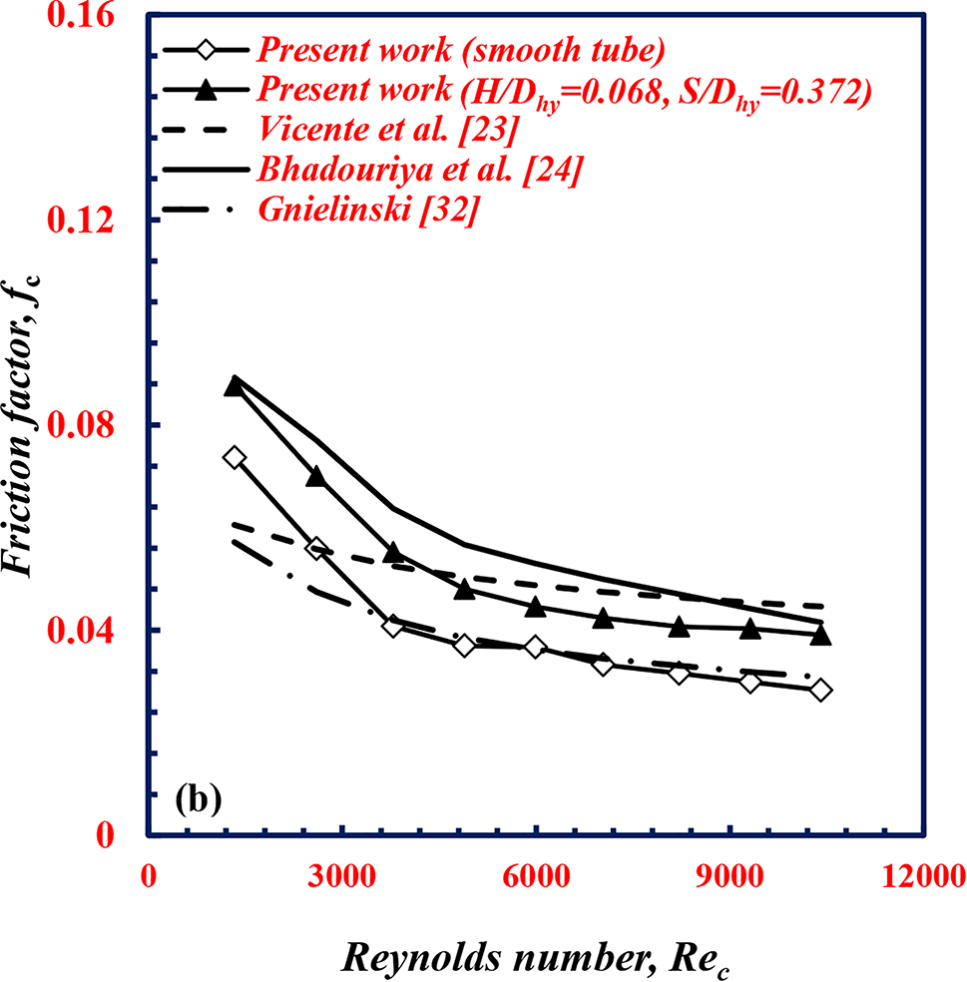
Validation of *f*_*c*_ with *Re*_c_ of twisted spiral tube.

The pressure data in terms of *f* are also validated by comparing the *f* of the cold water with the published correlations of Vicente et al.^23^ and Bhadouriya et al.^24^ for twisted tubes, and Gnielinski^32^ for smooth tube. As depicted in Fig. 21. The figure shows that the present data trend for the twisted corrugated tube agrees well with the correlations of Vicente et al.^23^ and Bhadouriya et al.^24^, with average deviations of 13.9%, and 15.6%, respectively, whereas the present results for the smooth tube presented lower values than the Gnielinski eq. with a variation of 4.7%.

**Table 3:** Correlations of the *Nu* and *f* for double twisted and smooth tubes.

Reference	correlation	Correlations limits	Eq. No.
Vicente et al.^23^	$$\begin{gathered} N{u_o}{\text{=}}=0.403{(Re - 1500)^{0.74}}P{r_o}{\,^{{\text{0.44}}}}{{\text{(}}H/{D_{hy,o}}{\text{)}}^{{\text{0.53}}}}{{\text{(}}S/{D_{hy,o}}{\text{)}}^{{\text{-0.29}}}} \hfill \\ {f_o}{\text{=}}=1.47*R{e^{ - 0.16}}{{\text{(}}H/{D_{hy,o}}{\text{)}}^{{\text{0.91}}}}{{\text{(}}S/{D_{hy,o}}{\text{)}}^{{\text{-0.54}}}} \hfill \\ \end{gathered}$$	= 2000 ≤ *Re* ≤ 90,000 and = 2.5 ≤ *Pr* ≤ 100	22
Bhadouriya et al.^24^	$$\begin{gathered} \frac{{N{u_{twisted}}}}{{N{u_{straight}}}}{\text{2.12}}R{e_o}^{{\text{0.06}}}\,Pr_{o}^{{0.6}}\,{{\text{(}}\frac{{4D_{{hy,o}}^{2}}}{{\pi d_{i}^{2}}}{\text{)}}^{{\text{0.05}}}}{{\text{(}}S/{D_{hy,o}}{\text{)}}^{{\text{0.08}}}} \hfill \\ \frac{{{f_{twisted}}}}{{{f_{straight}}}}{\text{5.8}}R{e_o}^{{{\text{-0.06}}}}{{\text{(}}\frac{{4D_{{hy,o}}^{2}}}{{\pi d_{i}^{2}}}{\text{)}}^{{\text{-0.2}}}}{{\text{(}}S/{D_{hy,o}}{\text{)}}^{{\text{-0.6}}}} \hfill \\ \end{gathered}$$	3000 ≤ Re ≤ 100,000; 10.6 ≤ $S/{D_{hy,o}}$ ≤ 15; 0.19 ≤ $\frac{{4D_{{hy,o}}^{2}}}{{\pi d_{i}^{2}}}$ ≤ 0.51	23
Gnielinski^32^	$$\begin{gathered} N{u_o}{\text{=}}\frac{{(f/8)\left( {Re - 1000} \right)Pr}}{{1+12.7\sqrt {f/8} \left( {P{r^{2/3}} - 1} \right)}} \hfill \\ {f_o}={\left( {1.82\log R{e_o} - 1.64} \right)^{ - 2}} \hfill \\ \end{gathered}$$	3000 ≤ *Re* ≤ 5 × 10^6^	24

## Correlations

New correlation for *Nu*_*o*_ and *f*_*o*_ for a double-tube heat exchanger with an inner twisted spiral tube in the range of Reynolds numbers from 5000 to 50000 and from 1400 to 10400 for both the inner tube and outer tube sides at different pitches (*S*) of 3.9, 5.2 and 8.2 mm corresponding to pitch ratios and at different pitch heights (*H*) of 0.6, 0.95, and 1.15 mm, respectively.

22 $$N{u_o}{\text{=0.0149}}R{e_o}^{{{\text{0.8}}}}P{r_o}{\,^{{\text{-1.687}}}}{{\text{(}}H/{D_{hy,o}}{\text{)}}^{{\text{-1.993}}}}{{\text{(}}S/{D_{hy,o}}{\text{)}}^{{\text{1.209}}}}$$

23 $${f_o}{\text{=1.63}}R{e_o}^{{{\text{-0.0262}}}}P{r_o}{\,^{{\text{-4.476}}}}{{\text{(}}H/{D_{hy,o}}{\text{)}}^{{\text{0.0938}}}}{{\text{(}}S/{D_{hy,o}}{\text{)}}^{{\text{0.43}}}}$$

**Fig. 22:**
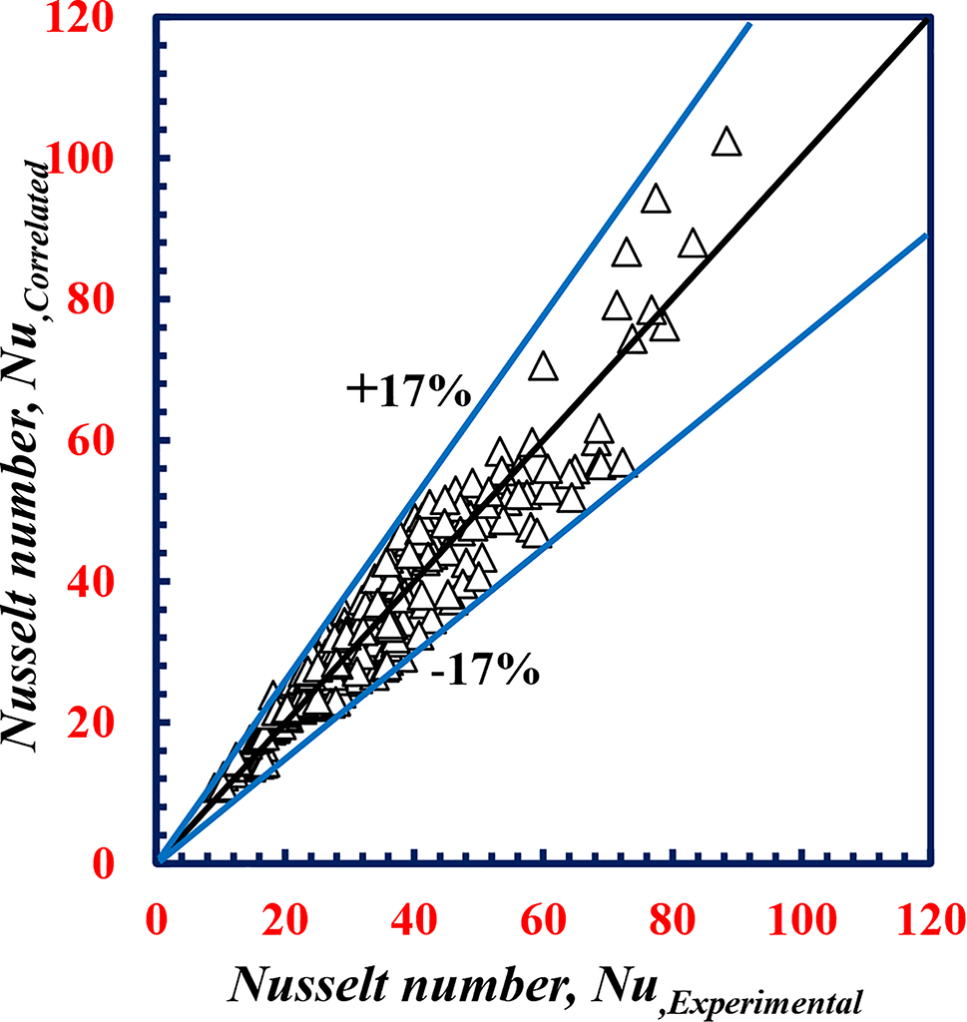
Variation between *Nu*,_*correlated*_ and *Nu*,_*experimental*_.

**Fig. 23:**
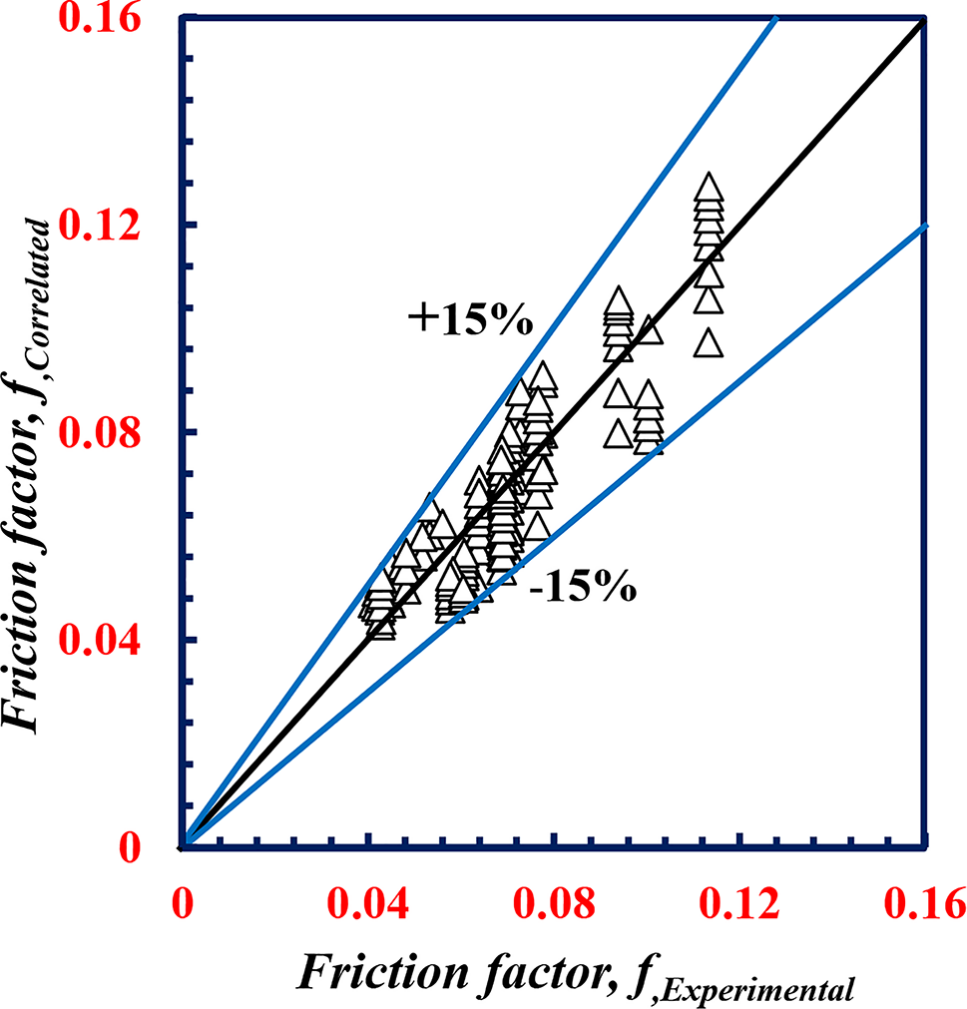
Variation between *f*,_*correlated*_ and *f*,_*experimental*_.

Equations 22 and 23 are varied, with maximum deviations of 17% and 15% for both *Nu*_*o*_ and *f*_*o*_, respectively, as shown in Figs. 22 and 23.

## Conclusion

The characteristics of fluid flow and heat transfer of a twisted spiral tube are compared with that of a conventional tube heat exchanger. The study investigates experimentally the effects of twisted spiral pitch ratio, *(S/D*_*hy*_*)* and twisted spiral ratio depth *(H/D*_*hy*_*)* on the thermal performance characteristics. The *Nu*, *f*, *Q/P.P*, *ε*, *ΔP* as well as *Re* were presented for all investigated parameters. The main conclusions can be summarized as follows:

- The twisted spiral tube improves the turbulence intensity of the fluid, which enhanced the heat transfer between the tube wall and the fluid by diminishing the thermal boundary layer.
- The *Nu*_*c*_ in the counter flow arrangement was 16% greater than that of the parallel flow arrangement under the same operating conditions.
- Both *H/D*_*hy*_ and *H/D*_*hy*_ plays a significant role on improving the thermal performance characteristics of the double tube heat exchanger.
- Increasing *S/D*_*hy*_ to 0.586 improve the *Nu*_*c*_ in the twisted spiral tubes is by 38% compared to the smooth tube at the expense of 33.2% increasing in *f*_*c*_ for the same flow conditions.
- Increasing *H/D*_*hy*_ to 0.082 enhances *Nu*_*c*_ by 44.9% compared to the smooth tube at the expense of 36.4% increase in the *f*_*c*_ for the same flow conditions.
- The maximum *η* reached 1.93 and 2.03 at *S/D*_*hy*_ of 0.278 and *H/D*_*hy*_ of 0.082, respectively.
- New correlations to predict *Nu*_*c*_ and *f*_*c*_ of the twisted spiral heat exchanger were correlated.

## Data Availability

The datasets generated during and/or analyzed during the current study are available from the corresponding author upon reasonable request.

## List of symbols

*A*: area, m^2^
*b*: twisted spiral tube side length, m
$\it C_{p}$: specific heat, kJ·kg^−1^·°C^−1^
*D*: Diameter ,m
$$\it D_{hy}$$: hydraulic diameter, m
*f*: friction factor
*H*: Depth oftwisted spiral tube,mm
*h*: convective heat transfer coeff.,W.m^−2^.°C^−1^
*k*: fluid thermal conductivity ,W·m^−1^·°C^−1^
*L*: Length, m
$\mathop m\limits^{.}$: mass flow rate, kg·s^−1^
*Nu*: Nusselt number
${\it \Delta\:P}$: pressure drop, Pa
*Pr*: Prandtl number, ($\it \mu$ ·*Cp*/*k*)
*Q*: heat transfer rate, W
*S*: pitch of twisted spiral tube, mm
$\it U_{\circ}$: overall heat transfer coeff.,W·m^−2^·°C−^1^
*T*: Temperature, °C
*V*: Velocity, m·s^−1^

## ***Greek symbol***

$$\epsilon$$: Effectiveness
$\eta$: Thermal performance criteria
$\mu$: dynamic viscosity ,kg·m^−1^·s^−1^
$$\rho$$: Density ,kg·m^−3^

## ***Subscripts***

*avg*: average
*b*: bulk
*c*: cold fluid
*h*: hot fluid
*i*: Inlet, inner
*LMTD*: log mean temperature difference, ^o^C
*max*: maximium
*min*: minimum
*o*: Outlet, outer
